# Leading Towards a Translation Readiness Framework: A Systematic Review of Deep Learning Approaches for Eye Disease Diagnosis

**DOI:** 10.3390/diagnostics16162500

**Published:** 2026-08-07

**Authors:** Usman Ali, Abdullahi Abubakar Imam, Rosyzie Anna Apong

**Affiliations:** 1 School of Digital Science, Universiti Brunei Darussalam, Tungku Link, Bandar Seri Begawan BE1410, Brunei; 2 Department of Information Sciences, University of Education Lahore, Vehari Campus, Vehari 61100, Pakistan

**Keywords:** retinal diseases, artificial intelligence, deep learning, diagnostic reliability, clinical validation, diagnostic imaging, systematic review

## Abstract

**Background:** Artificial intelligence has significantly improved the diagnosis of retinal diseases using fundus and optical coherence tomography. However, the pathway between the accuracy of the models and their clinical application is still unclear. **Objective:** The objective of this systematic review is to overcome this gap by correlating the quality of methodology with clinical preparation in the current studies and to critically examine the translation readiness possibility. **Methods:** The PRISMA-based review clearly addresses the six research questions in an organized manner. A comprehensive search is conducted across six electronic databases, resulting in a total of 883 records. After rigorous screening, a final set of 43 fundus- and OCT-based articles published between 2020 and August 2025 that passed a comprehensive eligibility criterion was selected. The selected studies are assessed using the custom AI-specific risk-of-bias assessment tool and the CLAIM checklist to measure the quality of technical reporting. The reviewer’s agreement is calculated using Cohen’s kappa, interrater reliability, and the intraclass correlation coefficient. **Results:** Although convolutional and hybrid deep-learning-based studies are often reported to have an accuracy of more than 95.0%, only 14.3% are truly clinically validated, and 23.1% do not include code or implementation data details. Reviewers’ agreement scores are from moderate to high (fundus-based ICC(2, 1) = 0.911, CI = [0.896, 0.923]) and (OCT based ICC(2, 1) = 0.643, CI = [0.422, 0.792]), supporting the reliability of the quality assessments. In order to combine these dimensions, we come up with a composite Technical-Clinical Integrity Index, which measures data transparency, rigor of validation, and reporting of performance. **Conclusions:** Retinal disease diagnosis has achieved high performance with low clinical transfer. This review provides the guidelines of a Translational Readiness Framework that can be used by researchers, clinicians, and health analytics teams to assess and implement credible artificial intelligence systems in ophthalmology.

## 1. Introduction

The retinal diseases are a primary reason behind preventable blindness, especially in the low- and middle-income areas that still lack specialist screening. It is estimated by the World Health Organization that more than 2.2 billion people in the world are visually impaired, with the leading cause of blindness due to diabetic retinopathy and glaucoma, which can be avoided in 80% of cases with early diagnosis and treatment [1]. In this regard, AI-enhanced screening and tele-ophthalmology network integration can make ophthalmologists experience better early detection, triage, and workload optimization, which later will make diagnostic services available to the remote population [2]. In addition to making work more accurate, AI has the capacity to transform clinical practice by putting more importance on the complicated cases that actually deserve specialist attention.

The spreading use of AI in eye image analysis has become one of the major technological changes in recent years. The new algorithms are detected, localized, and classified pathological retina features of Diabetic Retinopathy (DR), Glaucoma, Retinal Vein Occlusion (RVO), Choroidal Neovascularization (CNV), Macular Edema (ME), Age-related Macular Degeneration (AMD), as well as Retinal Vascular Analysis in both modes of fundus photography and OCT [3,4]. The detection of such conditions as early as possible is important because, in most cases, a late diagnosis usually results in permanent blindness [5].

**Figure 1:**
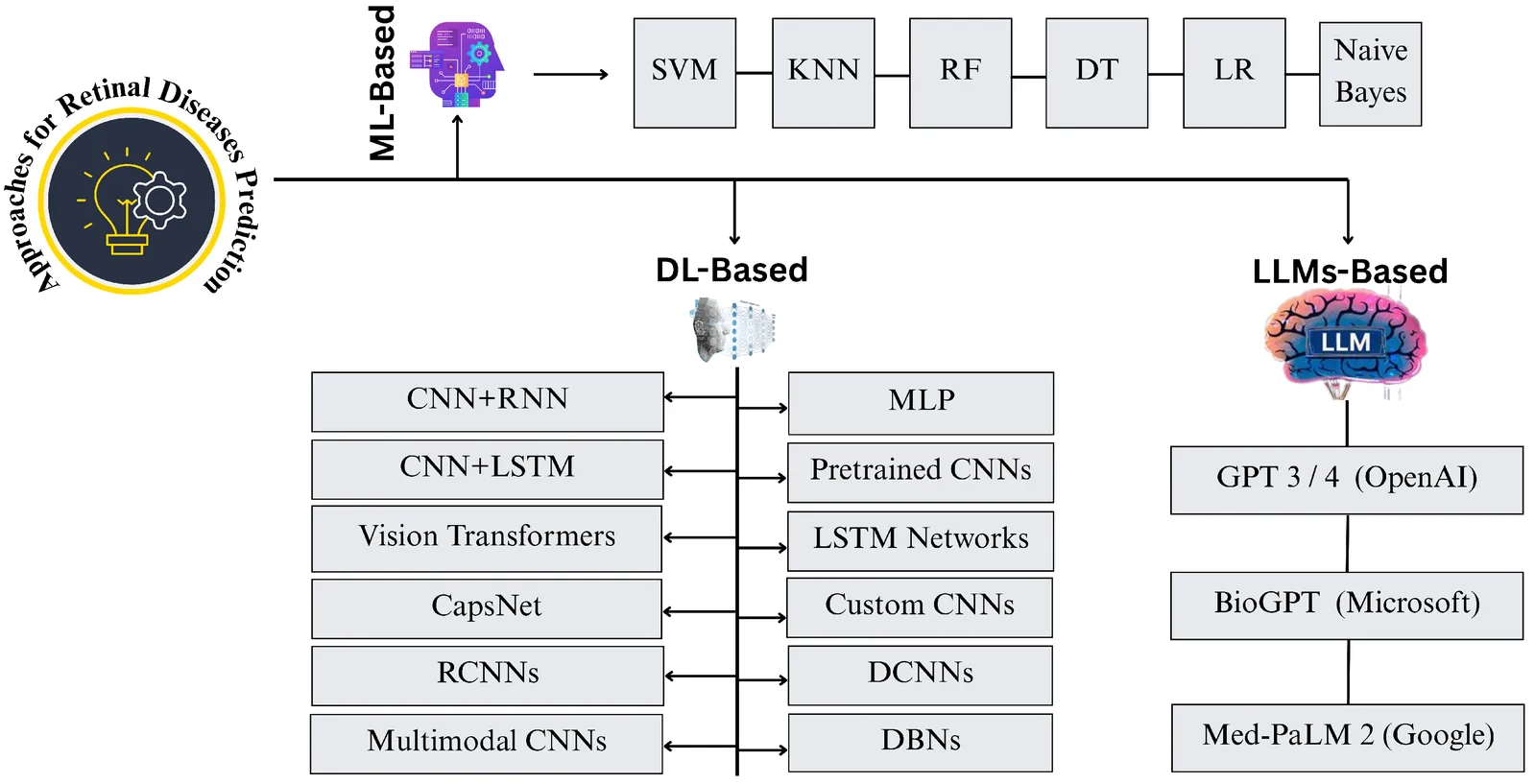
Approaches for retinal disease segmentation and classification.

Figure 1 provides a schematic view of the general steps taken to detect retinal disease using computational methods in the different imaging modalities.

In the past, studies in this field were conducted with classical machine-learning classifiers, including support vector machines (SVM), k-nearest-neighbors (KNN), decision trees, and logistic regression, where the feature extraction was done manually [6,7]. These techniques were good but needed fine-tuning by experts and had a poor scaling capability. The introduction of deep learning, specifically, convolutional neural networks (CNN) and hybrid neural networks, including CNN-LSTM, Vision Transformers, and Capsule Networks, made it possible to learn features directly on retinal images, end-to-end [8,9]. EfficientNet and ResNet pre-trained networks also increased small dataset accuracy, and attention architectures and explainable-AI tactics started to tackle the long-standing interpretability problem of clinical decision-making [10,11,12,13,14,15]. In spite of these developments, accurate and generalizable AI implementation in the field of ophthalmology is a difficult task.

### 1.1. Gaps and Motivation

Though many reviews have reported AI advancement in retinal imaging, the majority are descriptive, summing up constructs, dataset volumes, or performance indices, and do not question the fact that reproducibility, transparency, and real-life validation are poor. The dominant literature focuses on the accuracy and ignores the influence of methodological rigor on reliability and clinical deployment readiness [16,17,18,19]. This gap highlights that clinicians and data-science teams are yet to have a common framework for evaluating the transferability of the results of a model to patient-care settings, and also highlights the necessity of an analytical synthesis providing the connection between technical design decisions and their clinical and operational implications.

### 1.2. The Conceptual Framing and Methodological Novelty

In order to overcome these constraints, the present review incorporates several methodological points of view. It is a PRISMA 2020 systematic review framework [20], with two supplementary evaluation instruments: A custom AI-specific risk-of-bias (ROB) assessment and validation rigor on five generic domain items, and CLAIM to review technical rigor. Based on these tools, we propose a composite measure, the Technical-Clinical Integrity Index (TCII), to measure how data quality, the depth of the validation, and completeness relate. It makes the review less of a descriptive catalog, but rather of an analytical discussion of what factors are behind trustworthy AI in retinal imaging.

**Figure 2:**
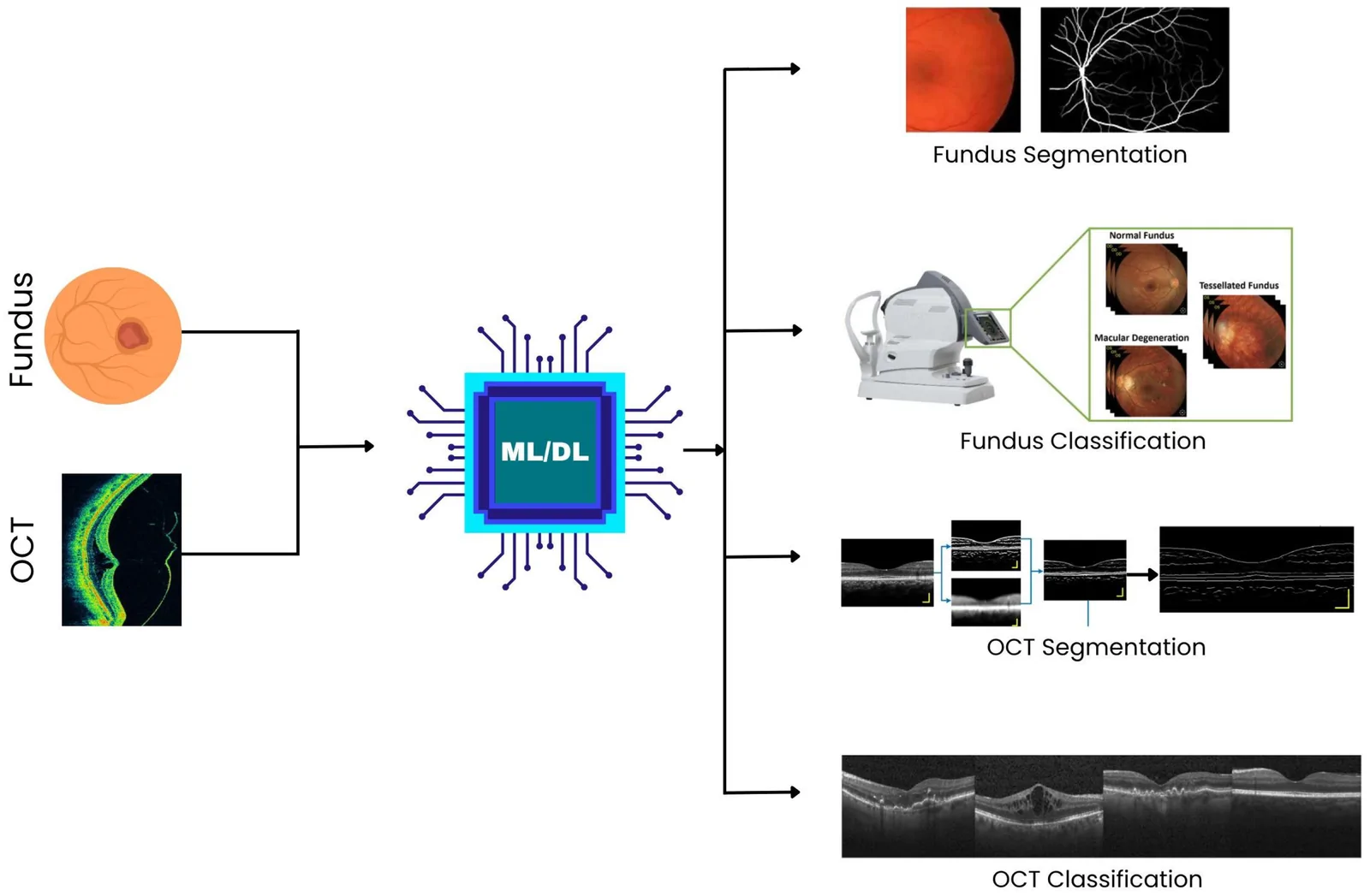
An overview of image modalities and the application of ML/DL techniques on each modality.

An overview of ML/DL techniques and their applications on selected modalities is shown in Figure 2.

### 1.3. Objectives and Analytical Outcomes

The main goal of the review is to build an integrative analytical framework that will associate technical performance with translational readiness in AI-based retinal diagnostics. The Research Objectives (RO’s) of this study are to answer the research questions, as outlined in Table 1.

### 1.4. Article Structure

The rest of this manuscript is structured in the following way. In Section 2, the review protocol, search strategy and quality assessment methods are described according to PRISMA 2020. Section 3 gives descriptive and analytical results of fundus and OCT studies, whereas in Section 4, the interpretive insights are discussed and the proposed Translational Readiness Framework is presented. The final sections are summative in terms of important implications, limitations and future research and clinical adoption directions.

## 2. Materials and Methods

### 2.1. Protocol and Registration

The review protocol was retrospectively registered and updated with the Open Science Framework (OSF) to improve transparency and reproducibility. (Registration DOI: https://doi.org/10.17605/OSF.IO/ZBY9H).

This study followed PRISMA (2020) guidelines and search strategies used in fundus and OCT syntheses to define our keyword set and inclusion window [20]. A metadata file was created for more than twenty parameters from the fundus and OCT-based studies. Then, the most important parameters were screened out from the metadata to carry out further analysis of risk of bias and compliance percentages, and the Checklist for Artificial Intelligence in Medical Imaging (CLAIM) tool, [21,22]. While these frameworks provided valuable guidance, we did not rigidly apply every checklist item. Instead, we used the components that aligned best with the goals of our review and interpreted them based on our current level of expertise and the characteristics of the included studies. Based on different parameters like frameworks used, evaluation techniques, datasets, clinical trustworthiness, and code availability, etc., a comprehensive comparison was done in both visual and tabular formats.

**Figure 3:**
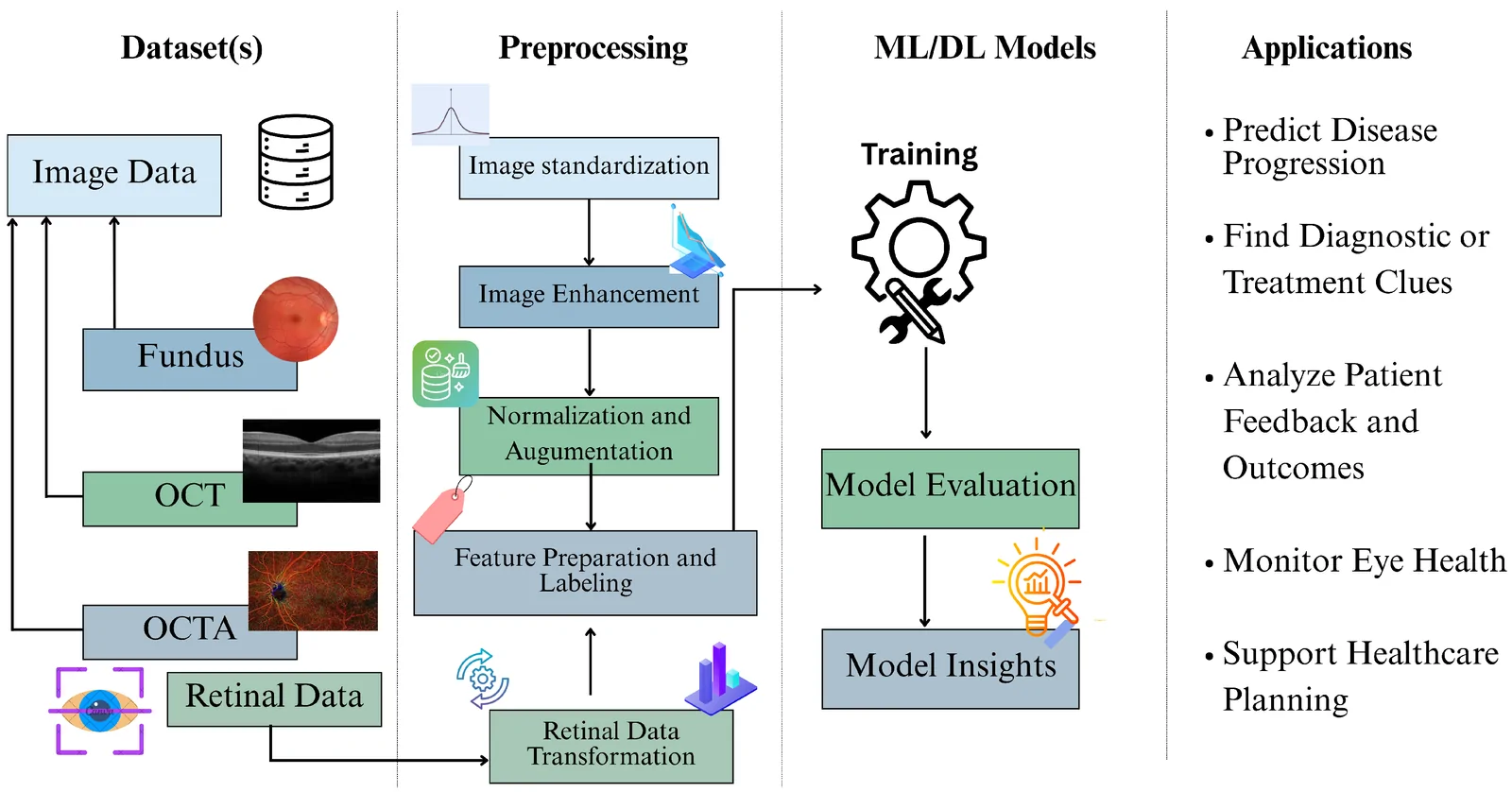
A generalized working mechanism of ML/DL models for eye disease classification across modalities.

Figure 3 outlines the working mechanism of the studies when they use deep learning to detect retinal diseases from fundus or OCT images. It usually starts with collecting image data, either from hospitals or public datasets such as EyePACS and APTOS [23,24]. These images often differ in quality, resolution, and labeling, so they need to be cleaned up before any model training starts. That is where preprocessing comes in practice; researchers typically resize the images, adjust brightness or contrast, filter out noise, normalize pixel values, or apply data augmentation. These steps help to make the data set more consistent and improve the model’s ability to generalize to new samples [25]. After that, labels are applied, and features are either extracted manually (in older studies) or automatically learned through deep learning models. The most recent works have used CNNs, ResNets, or sometimes even Vision Transformers directly on the processed images. But the goal isn’t just to get high accuracy. Many of these models are designed to help predict disease stages, detect early signs, or track the progression levels of diseases. A few studies have looked at how these tools could support public health or hospital planning by analyzing large datasets for trends in eye health across populations [26,27].

This systematic review analyzed data from previously published studies only and did not involve human participants or identifiable personal data; therefore, Institutional Review Board (IRB) approval was not required. Furthermore, according to institutional research ethics policy and international guidelines, including the Declaration of Helsinki, systematic reviews of the published literature do not require IRB or Ethics Committee approval; also, a complete meta-analysis was not carried out. The main reason is that the included studies were all over the place in terms of datasets, metrics, and even how the models were tested. Most used accuracy, others preferred AUC or sensitivity, and a few had their own custom thresholds [28,29,30,31,32,33].

### 2.2. Eligibility Criteria

A systematic eligibility (inclusion and exclusion) criterion is defined, according to which full-text studies from reputable journals, conferences and venues are included. The detailed criterion for this review is given in the following Table 2.

The restriction to English-language publications and single-modality studies was applied to maintain methodological consistency and ensure reliable comparison across included works. English-language filtering was necessary as all reviewers were proficient in English, enabling accurate interpretation of technical and clinical reporting. Similarly, limiting the review to single-modality (fundus or OCT) studies reduced heterogeneity arising from multi-modal fusion strategies, which often involve differing data distributions, architectures, and validation protocols.

However, these criteria may introduce selection bias. Excluding non-English studies could omit relevant contributions from regions actively developing ophthalmic AI systems. In addition, the exclusion of multi-modal approaches may limit the generalizability of the findings, as such systems are increasingly explored for improving diagnostic performance in clinical settings. These factors are considered when interpreting the scope and applicability of the results.

Additionally, we applied a PICO strategy [34] as shown in the Table 3.

### 2.3. Search Strategy and Selection Process

The literature search was designed in accordance with PRISMA 2020 recommendations to ensure transparency, reproducibility, and methodological rigor. A comprehensive and database-specific search strategy was developed in consultation with established practices in medical imaging and artificial intelligence reviews. Searches were conducted across six major bibliographic databases: PubMed (MEDLINE) [35], IEEE Xplore [36], Web of Science Core Collection [37], Scopus [38], Semantic Scholar [39], and Google Scholar, supplemented by manual reference screening of eligible articles.

For PubMed, controlled vocabulary terms (Medical Subject Headings, MeSH) [40] were combined with free-text keywords to capture both indexed and recently published records. For engineering-focused databases such as IEEE Xplore and Scopus, equivalent keyword-based strategies were adapted to platform-specific query syntaxes. The search targeted studies applying deep learning techniques to single-modality retinal imaging, specifically fundus photography or optical coherence tomography (OCT).

To support full reproducibility, the complete and verbatim search strings for each database, including Boolean operators, field tags, applied filters, and the exact dates of the final searches, are provided in Appendix A.

Given the large volume of records retrieved from multiple bibliographic sources, a hybrid screening strategy combining automated prioritization with independent human review was adopted. This approach was designed to improve screening efficiency while maintaining adherence to PRISMA 2020 recommendations for transparency and reproducibility.

In the first phase, title and abstract screening was supported by three widely used automation-assisted tools: Rayyan [41], Elicit.ai [42], and Bohrium [43]. All retrieved records were uploaded into each platform independently. These tools generate relevance scores based on semantic similarity between article content and predefined inclusion criteria. Scores ranged from 0 (least relevant) to 100 (most relevant), reflecting the estimated likelihood that a record met the eligibility criteria.

A relevance threshold of 70 was applied to prioritize records for human review. This cutoff was selected following a pilot calibration exercise conducted on a random subset of 100 records, during which multiple threshold values (60, 70, and 80) were tested. A threshold of 70 was found to balance sensitivity and screening burden, minimizing false exclusions while substantially reducing the number of records requiring full manual inspection. Records scoring at or above this threshold in at least one tool were advanced to the next stage.

Records scoring below 70 across all three tools were provisionally excluded. To safeguard against false-negative exclusions, a random sample comprising 20% of these provisionally excluded records was independently re-screened by human reviewers. This verification step confirmed that no eligible studies were systematically excluded by the automation-assisted filtering.

In the second phase, all records advanced by the automated tools were screened independently by two reviewers at the title and abstract level. Reviewers worked in parallel and were blinded to each other’s decisions. Disagreements occurred in approximately 12% of screened records at this stage and were resolved through discussion. If consensus could not be reached, a third reviewer adjudicated the decision.

Full-text screening was subsequently conducted independently by the same two reviewers for all records meeting the title and abstract inclusion criteria. Discrepancies at the full-text stage were infrequent (less than 10%) and were resolved using the same consensus-based procedure. Reasons for exclusion at the full-text stage were documented and reported in the PRISMA flow diagram.

This structured integration of automation-assisted ranking with duplicate independent human screening ensured both efficiency and methodological rigor, while maintaining full traceability of decisions at each stage of the selection process.

The automation-assisted screening tools were not used as standalone decision-makers but as prioritization aids. A record was retained for human review if it met the relevance threshold in at least one platform, thereby reducing the risk of tool-specific bias. The 20% manual re-screening of provisionally excluded records was performed using random sampling without replacement, and all such records were assessed independently by two reviewers following the same eligibility criteria applied to the main screening cohort. Steps-wise attribution of screening stages between automation tools and human reviewers is shown in Table 4.

### 2.4. Data Extraction

The last stage was the extraction of data using a structured process after the inclusion of the eligible studies to guarantee consistency, accuracy, and reproducibility. A data extraction form was designed using Microsoft Excel in order to identify methodological, technical, and reporting-related aspects of each study. The form had over twenty predefined parameters, which included publication information, dataset properties, imaging modality, model architecture, preprocessing options, validation scheme, explainability analysis, reproducibility measures, and reported performance measures. To determine the clarity, completeness, and appropriateness of the data extraction form to the review objectives, the data extraction form was first piloted on a sub-sample of five randomly selected studies before full-scale extraction commenced. This pilot phase was used to make minor adjustments to the parameter definitions and coding rules in order to reduce ambiguity in future extraction.

Two reviewers performed data extraction for all the studies that were included. The reviewers were filling in the extraction form with no direct access to each other. The data that was extracted was then compared, and discrepancies were detected using automated cross-tabulation in the spreadsheet. All disputes were solved by discussion, and in case of disagreement, to come to a consensus, a third reviewer checked the initial publication and decided on the final verdict.

To minimize transcription errors and subjective interpretation, it was done with a dual independent extraction process that is facilitated with pilot testing, and explicit resolution rules to enhance the methodological reproducibility of the review.

### 2.5. Risk of Bias and Quality Assessment

Two tools were used in evaluating the custom AI ROB assessment and the quality of the chosen studies, namely Robvis generic, which is a visualization tool and CLAIM. These tools are widely applied in the visualization of ROB and checking technical quality, respectively. Table 5 presents the five domain items (D1–D5) selected. The domains on each of these tools were rated by two reviewers on the final selections. A third reviewer was used to cross-check in case of any disagreement on a domain between two reviewers.

To avoid ambiguity with established risk-of-bias instruments, this rubric is hereafter referred to as a *custom AI-specific risk-of-bias (ROB) assessment*. While the proposed five-domain ROB framework captures key aspects of transparency, validation, and reproducibility, it does not explicitly model certain bias sources such as labeling quality, patient selection bias, or subtle forms of data leakage beyond preprocessing reporting. These elements were partially inferred through domain definitions (e.g., preprocessing and dataset description) but were not independently scored due to inconsistent reporting across studies. Each domain was assessed using predefined qualitative criteria aligned with PROBAST-AI guidance [44], with calibration performed among reviewers prior to scoring. The tool was intentionally designed as a focused, interpretable subset of broader AI-specific bias considerations rather than a comprehensive replacement for established instruments.

**Figure 4:**
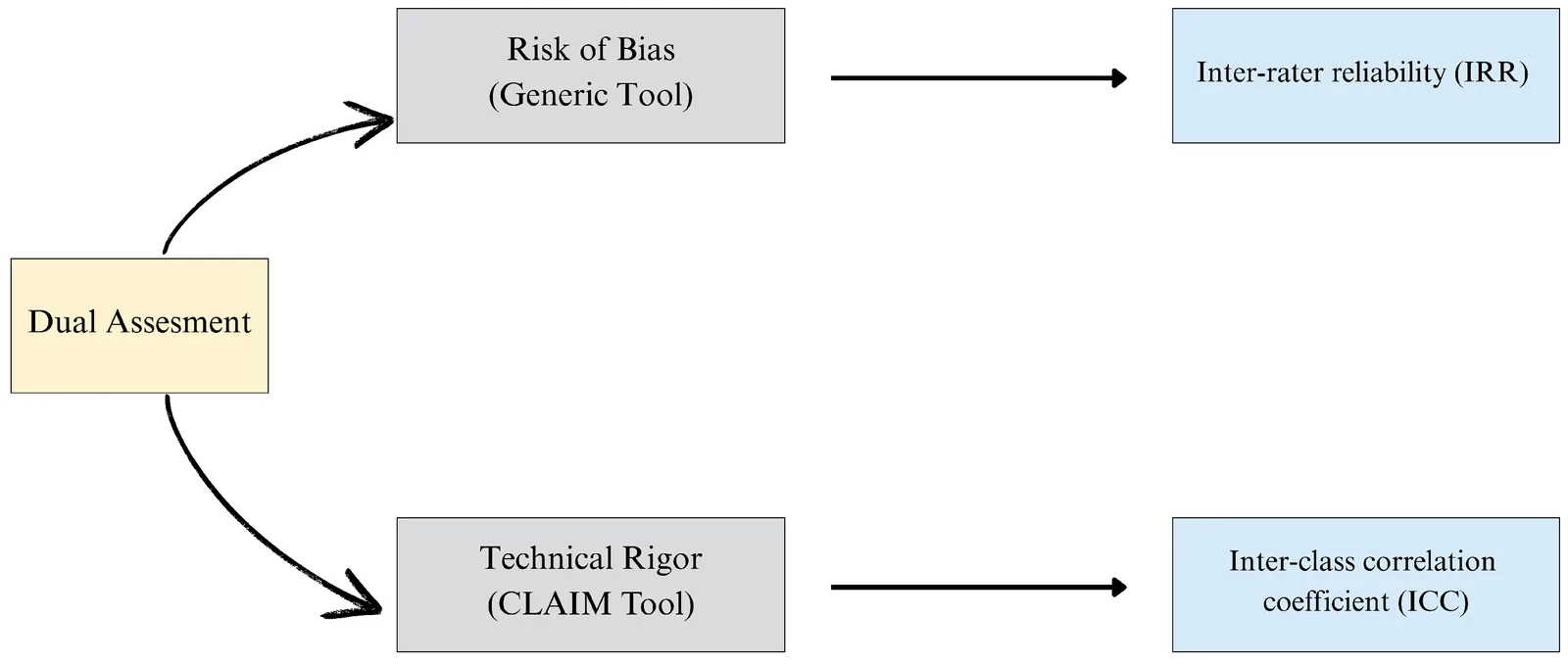
Workflow diagram of dual assessment process.

For the visualization of the results, the ROB Visualization Tool (ROBVIS) was used [21]. The workflow diagram is shown in Figure 4.

The calculation of ROB in relation to our custom AI tool and domain ratings with respect to signaling questions is performed in a systematic way, as shown in Table 6.

#### Statistical Analysis

Interrater agreement for study selection and data extraction was carried out through Cohen’s kappa “*k*” [45] and interclass correlation coefficient (ICC) [46]. The mathematical formulas for each measure are provided below. The formula for Cohen’s Kappa (unweighted) is shown in Equation (1)

(1) $$k=\left(P_{o}-P_{e}\right)/\left(1-P_{e}\right)$$

where:

$P_{o}$ = observed agreement;

$P_{e}$ = expected chance of agreement.

The inter-class correlation coefficient (ICC) for absolute agreement as a two-way random effect model is shown in Equation (2)

(2) $$ICC\left(2,1\right)=\left(MS_{r}-MS_{e}\right)/\left(MS_{r}+\left(k-1\right)MS_{e}\right)$$

where:

$MS_{r}$ = mean square between subjects;

$MS_{e}$ = mean square error;

*k* = No. of raters/repeated measurements.

These statistical tests were used to confirm that the reviewers’ decisions were consistent and reliable. The agreement values were checked before moving forward with the primary analysis.

The domain-level ratings obtained from *custom AI-specific (ROB) assessment* (bias and validation rigor), CLAIM (reporting transparency and reproducibility), were later merged into one composite indicator called Technical–Clinical Integrity Index (TCII), as described in the later subsection.

### 2.6. Technical–Clinical Integrity Index (TCII) Computation

To allow the unified comparative analysis of methodological robustness of the studies, we created a composite measure of the methodological robustness termed the Technical-Clinical Integrity Index (TCII). The index was developed to measure the combined impact of three key methodological dimensions, dataset integrity, validation rigor, and reporting transparency on the credibility and clinical readiness of each AI model. Each included study was given a normalized sub-rating (between 0 and 1) for the following components:

- Dataset quality (Dq = D1 + D2): Completeness, data diversity, public availability, and preprocessing steps;
- Validation strategy (Vs = D4): Presence of external/clinical validation, cross-validation folds, and sample independence;
- Transparency and reproducibility (Tr = D3 + D5): Being able to provide source code or pretrained models, being able to define performance measures.

The total TCII was calculated as a weighted sum of these three components with the following formula:

(3) TCII=0.35(Dq)+0.40(Vs)+0.25(Tr)

The selection of the weighting scheme was based on pilot testing and discussion among two independent reviewers, based on validation rigor as the most important factor in determining translational reliability. All variables were rated using explicit criteria that were derived from *custom AI-specific (ROB) assessment* and CLAIM domains, with any disagreement being settled by consensus with the third reviewer. TCII values that are near to 1 indicate high-quality technical and high clinical applicability studies [16,47].

To find out the correlation between TCII ratings and reported diagnostic performance parameters (accuracy and AUC), non-parametric correlation tests (Spearman’s rho) and descriptive statistics (mean, SD and interquartile range) were then used to explore the correlation between TCII ratings and the diagnostic performance parameters. The weighting scheme was established by expert opinion and pilot testing, and set more weight on validation rigor because one knows the significance of validation rigor in clinical translation. This is, however, a method that is subjective in nature and not empirically optimized. Also, other dimensions like explainability (XAI) and regulatory preparedness were not explicitly included in the TCII formulation because of the inconsistent reporting of the studies. These aspects are becoming critical to the real-world implementation and must be taken into account in further improvements of the index.

To test the strength of TCII-based inferences, a sensitivity analysis was performed, where each weight was changed by ±0.10, and the rest of the elements were changed correspondingly to maintain a unit-sum constraint. In all the tested setups, the relative rankings of studies and the found associations between TCII and the reported diagnostic performance measures were relatively consistent, and no significant variations to the direction or significance of correlations were found. This indicates that the interpretive conclusions made by TCII are not too sensitive to small changes in the weighting scheme. The TCII construct can be improved in the future by data-based weighting schemes or expert opinion, especially as ophthalmic AI reporting standards and practices become more mature.

### 2.7. Translational Readiness Framework (TRF) Development

Building on the TCII analysis, a structured, threshold-informed Translational Readiness Framework (TRF) was established to map the translation of technical integrity into the potential clinical implementation.

The framework outlines three stages of increasing state readiness:

- Data Integrity: training data completeness and diversity, transparent preprocessing, and bias control;
- Validation Rigor: strength of internal and external validation, including independent dataset testing;
- Clinical Deployment Readiness: availability of interpretability mechanisms, clinician-in-the-loop validation, and demonstration of reproducibility in a real-world setting.

The TRF thus operationalizes the results of TCII into a decision-support structure that can be used to guide researchers, clinicians, and teams of health analytics professionals in determining whether or not an AI model is technically sound and practically implementable. The position of each study within the TRF was qualitatively assigned in accordance with the TCII percentile and descriptive reporting characteristics.

To improve operational interpretability, indicative thresholds were aligned with TCII ranges such that studies with TCII ≥ 0.70 were primarily mapped to Data Integrity, progression toward Validation Rigor required evidence of external or multi-center validation, and Clinical Deployment Readiness was assigned only when studies demonstrated elements of real-world evaluation or clinician integration. While these thresholds are heuristic and derived from observed reporting patterns, they provide a structured approximation of translational progression.

Furthermore, the TRF is conceptually aligned with established guidance frameworks such as CONSORT-AI and SPIRIT-AI, particularly in relation to reporting transparency and validation practices [48]. However, formal regulatory pathways (e.g., FDA Software as a Medical Device guidelines) and deployment-specific requirements were not explicitly modeled, as such information was inconsistently reported across the included studies.

## 3. Results

### 3.1. Study Selection

**Figure 5:**
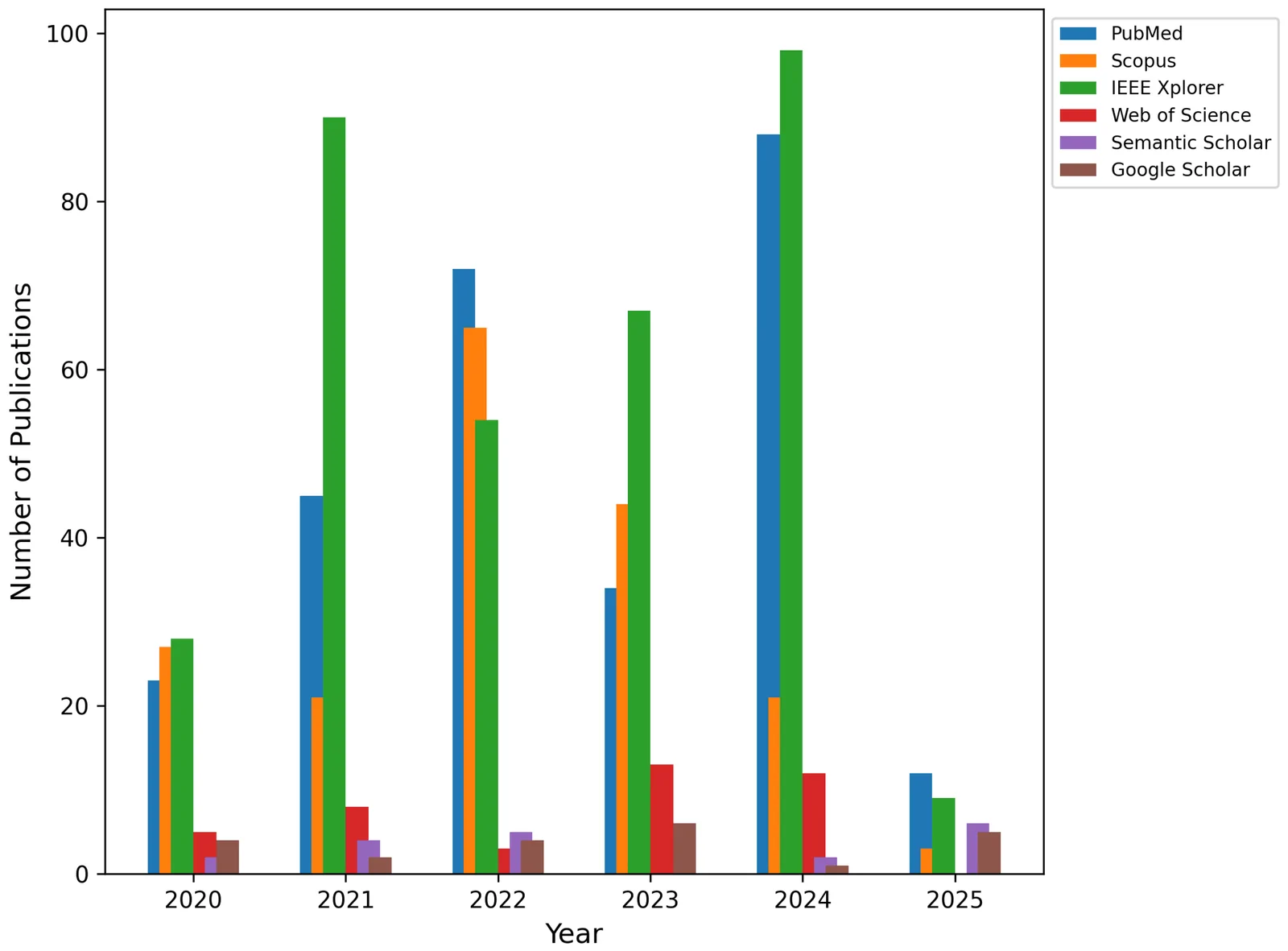
Overview of the literature searched through databases and search engines.

The study selection process for this review is based on two significant steps: the search through databases and registers, and the search through other methods (websites, etc.). We identified eight hundred and one (801) fundus and OCT-based records from the databases and registers, and eighty-two (82) most relevant records from other methods, eight hundred and eighty-three (883) in total. The details of databases and search engines used with the timeline are shown in Figure 5.

**Figure 6:**
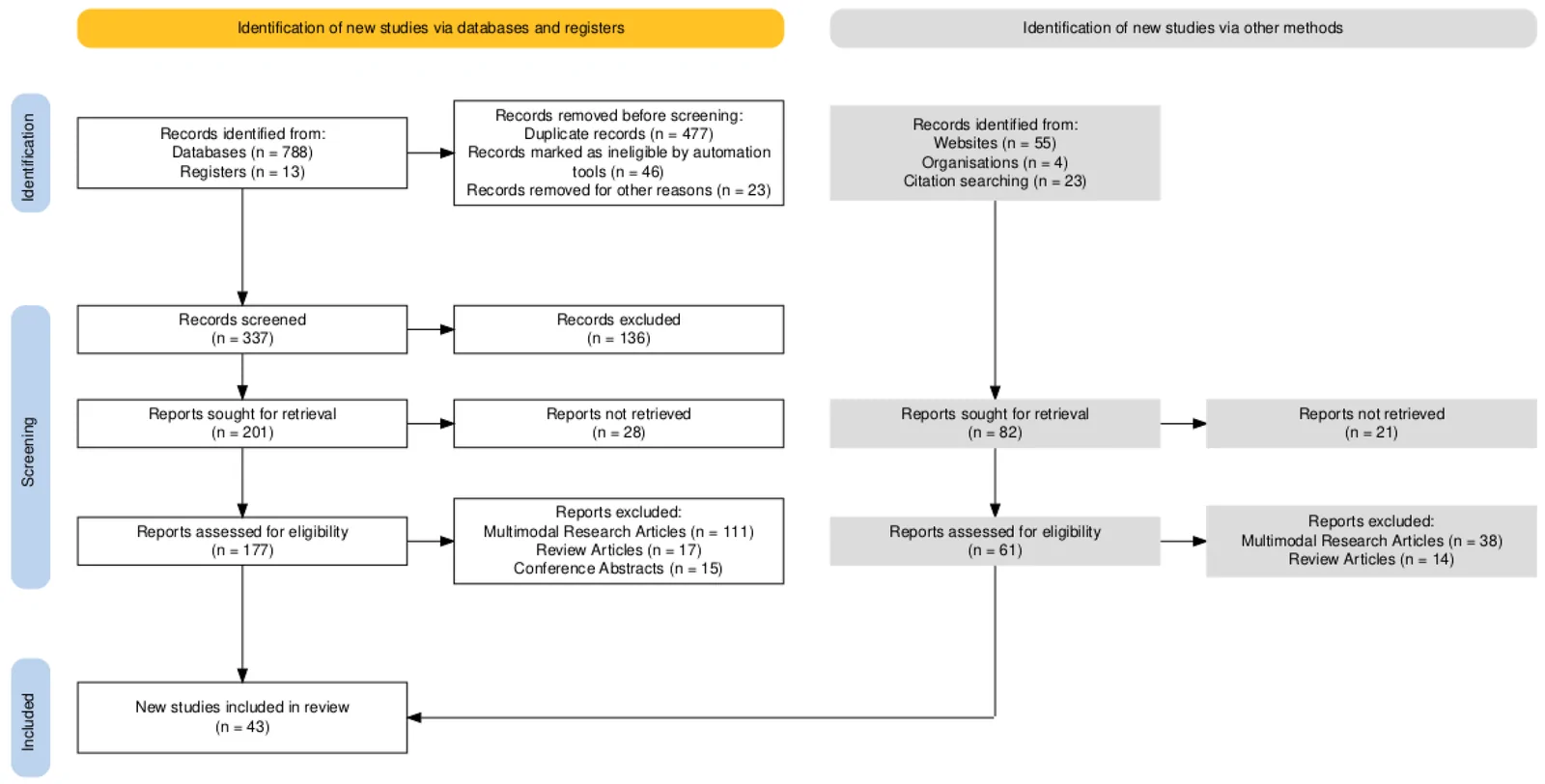
PRISMA flow diagram for identified searches of studies via databases and other methods.

A total of 546 records were removed prior to screening due to duplication, automation tool ineligibility, or other reasons. Through a rigorous screening process, 337 records were received, 136 were excluded from the screened records, 201 reports were sorted for retrieval and 177 reports were assessed for eligibility. Finally, 43 studies were included in the review. The overall screening procedure according to the defined inclusion and exclusion criteria is shown in Figure 6.

The diagram is created for the PRISMA flow diagram generator, estech.shinyapps.io [49], by inserting collected information. Out of 43 studies that meet the inclusion criteria, 20 studies are based on fundus image datasets and 23 on OCT datasets for eye disease classification and detection tasks. Because they used different imaging data, performance measures, and model setups, the results are shown in a narrative format rather than as a single combined figure. Key observations are supported with summary tables, bar plots, and risk-of-bias assessments, allowing for both qualitative interpretation and cross-study comparison. Agreement statistics, including Cohen’s kappa and intraclass correlation coefficients with corresponding confidence intervals, are provided where applicable to quantify the reliability of the reported measures.

#### 3.1.1. Studies Based on Fundus Image Datasets

Most of the literature work done on fundus image datasets was critically analyzed by the automation tools through a comprehensive screening strategy and cross-checked by the human reviewers to avoid any risk of bias. The literature is based on the most relevant features selected for the AI-specific domain items and CLAIM compliance. Most of the essential features, like augmentation/normalization strategies, multi-diseases support, code and data availability, were overlooked in previous reviews [25,50]. A comparative summary of included studies based on fundus images is shown in Table 7.

#### 3.1.2. Studies Based on OCT Image Datasets

The studies based on OCT images were also filtered out through a two-step rigorous screening process, as discussed in the previous sections. The feature selection was quite similar to that of the fundus images to meet the requirements for OCT studies. Twenty-three selected studies in the OCT domain with the sorted parameters are shown in Table 8.

#### 3.1.3. Framework Specific Insights

**Figure 7:**
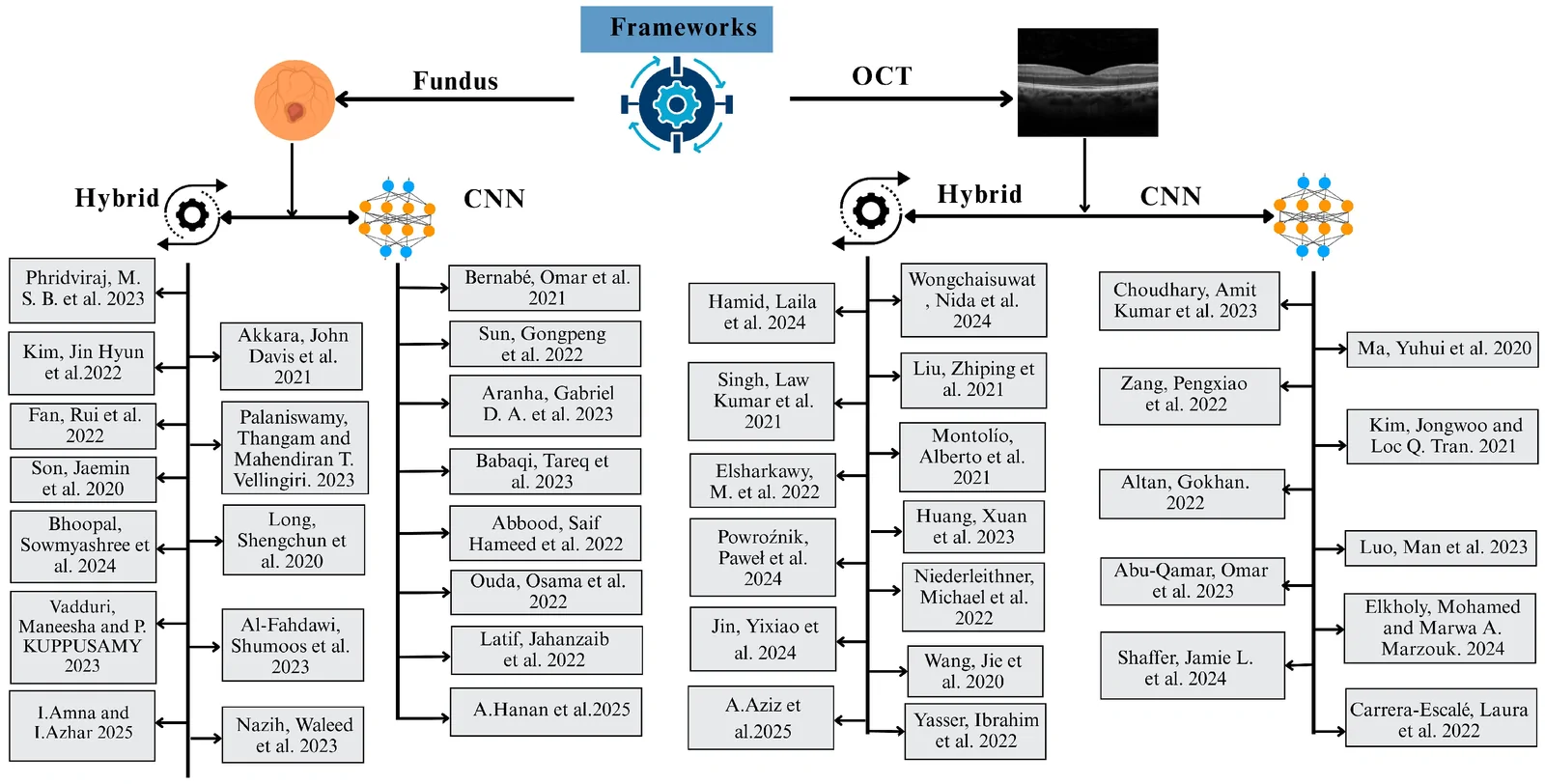
Frameworks used by the researchers in studies under consideration.

Figure 7 reflects how researchers have used different models to process fundus and OCT images. On the fundus side, most studies stuck with CNNs; they have been a solid go-to for identifying diabetic retinopathy, glaucoma, and AMD. Studies like those by [53,54,55,60,61,65,66,69] showed how CNNs can do a decent job learning features on their own, without much human help. They worked across different datasets, too, which made them useful for broader testing. But not all stopped there. Some researchers tried mixing CNNs with other models, such as using SVMs, attention layers, or LSTMs, to achieve better results. For instance, refs. [8,49,51,56,57,58,59,62,63,68] went with hybrid designs that helped especially when the data was imbalanced or tricky. There were also cases where researchers blended manual features with deep ones. Refs. [64,67] did that, and their models seemed better at catching early signs that might otherwise be missed.

OCT-based studies took a slightly different path. CNNs were still widely used, but the goals were often more about structure, such as segmenting retinal layers, rather than just classification. Researchers such as [33,71,74,75,77,78,79,83,85] have customized their networks to better fit OCT data, achieving solid accuracy in detecting diseases like AMD or macular edema. Others pushed the design further with tricks like image enhancement, transfer learning, or model ensembles; refs. [31,81,84] all went that route. Their setups weren’t just about accuracy; they tried to create models that could handle messy images or subtle features and still yield results that made sense to clinicians.

A noticeable shift in recent work is the gradual adoption of Vision Transformer (ViT) architectures and their hybrids with CNNs, enabling the capture of long-range spatial dependencies in retinal images. These architectures have demonstrated promising results, particularly for fine-grained lesion localization in diabetic retinopathy and macular degeneration [29,30,72,73,76,82]. Several studies also reported performance gains when pretraining these models on large-scale, non-medical image corpora followed by domain-specific fine-tuning. Despite their potential, adoption remains relatively low, partly due to the increased computational demands and scarcity of large annotated datasets [28,32,70,80,86].

#### 3.1.4. Dataset Specific Insights

**Figure 8:**
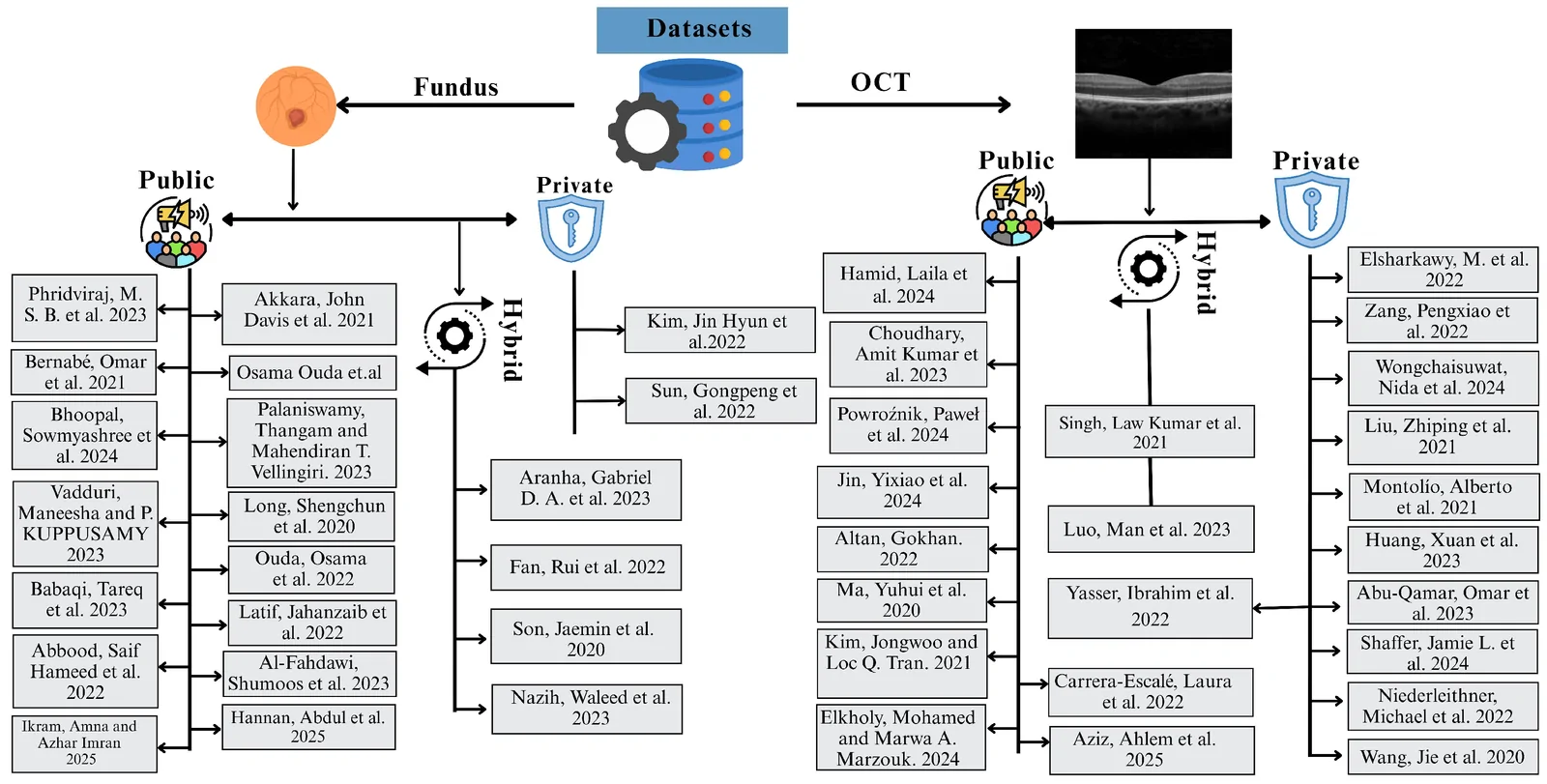
Datasets used by researchers in the studies under consideration.

Figure 8 also breaks down how studies have used different types of datasets, public, private, or a mix of both, depending on whether they worked with fundus or OCT images. For fundus-based research, a lot of work relied on public datasets like [23,24,87,88,89,90,91,92,93]. These datasets are widely used because they’re accessible and help researchers compare results across studies. Work by [51,53,58,59,60,61,62,63,64,65,66,67] followed, training deep learning models to detect diseases such as glaucoma and diabetic retinopathy. These public datasets made it easier to build models that generalize well.

On the other hand, some researchers used private data from hospitals or clinics. For instance, refs. [51,54] used private fundus images, which gave them more control over the data but also introduced challenges like variation in quality or label accuracy. Then there are hybrid approaches [55,56,57,68] that combine both public and private sources to boost model performance and make results more adaptable across different populations.

For OCT images, the pattern is similar. Studies like those [28,29,70,71,75,78,79,85,86] used public OCT datasets like those from [13,76,94,95,96,97,98,99]. These were mainly used for tasks like retinal layer segmentation or detecting fluid buildup, which are essential for diagnosing things like AMD or DME. Meanwhile, refs. [30,31,32,33,73,74,76,77,80,81,82] worked with private datasets collected in clinical settings. These gave more realistic conditions for testing their models, though the data often required extra preprocessing. Refs. [72,83] explored hybrid setups, using both public and private OCT data. They often added transfer learning or ensemble methods to handle differences in imaging quality and improve model accuracy in real-world cases.

### 3.2. Risk of Bias (ROB) Analysis

We used all fundus-based and OCT-based studies to analyze ROB across five domain items (D1 to D5) using predefined evaluation criteria of *custom AI-specific (ROB) assessment*. According to this criterion, for a given study, any domain out of five with a high ROB will contribute to the overall high ROB, and the same applies to a domain showing moderate risk [44].

The domain-wise results are shown in the combined summary Table 9. From which we can observe that code availability and clinical validation are the most scarce domains in fundus-based AI studies. In contrast, most of the studies have explicitly used diverse types of open-access and private fundus datasets and readily described the performance matrices used and their results.

The domain items D1, D2, and D5 have shown low risk across most of the studies, with overall ratings 65%, 95% and 75% respectively. This indicates that most studies have provided dataset availability, clearly defined preprocessing techniques, and performance measures used in their analyses, except for [51,52,53,54,56,57,63,66,67]. However, the other domain items have shown considerable gaps, like for D3, only two studies have provided full access to code for reproducibility [52,56]. Almost all other studies have not given any information regarding this domain, or high risk [62]. Likewise, in D4, more than half of the studies didn’t adequately describe the clinical validation process, leading to moderate risk; two of these applied real-time clinical testing in their research [61,62], and two have no information about the application of clinical validation [8,64].

OCT-based studies also lag in two domain items, D3 and D4. Recent studies in OCT-based image analysis have readily shown the performance measures of the applied models. Still, most of the studies lack reproducibility and clinical validations [28,29,30,31,33,70,71,72,73,74,75,76,77,78,79,80,81,82,83,84,85,86] as in the combined summary of the Table 9. The total of twenty-three included OCT-based studies is shown. OCT-based studies have shown a 47.9% low ROB for D1 and rated moderate risk or no information for these [29,31,32,33,73,74,77,78,80,81,82,83] studies. Similarly, 65.2% studies have low ROB for D5. However, it varies across other domains, like for D2, it is 82.6% low risk. In the case of D3, 65.2% studies provided no information. For D4, most of the studies have shown moderate risk and no information [28,29,30,31,33,70,71,72,73,74,75,76,77,78,79,80,81,82,83,84,85,86], except the study [32] shows low risk for this domain item.

**Figure 9:**
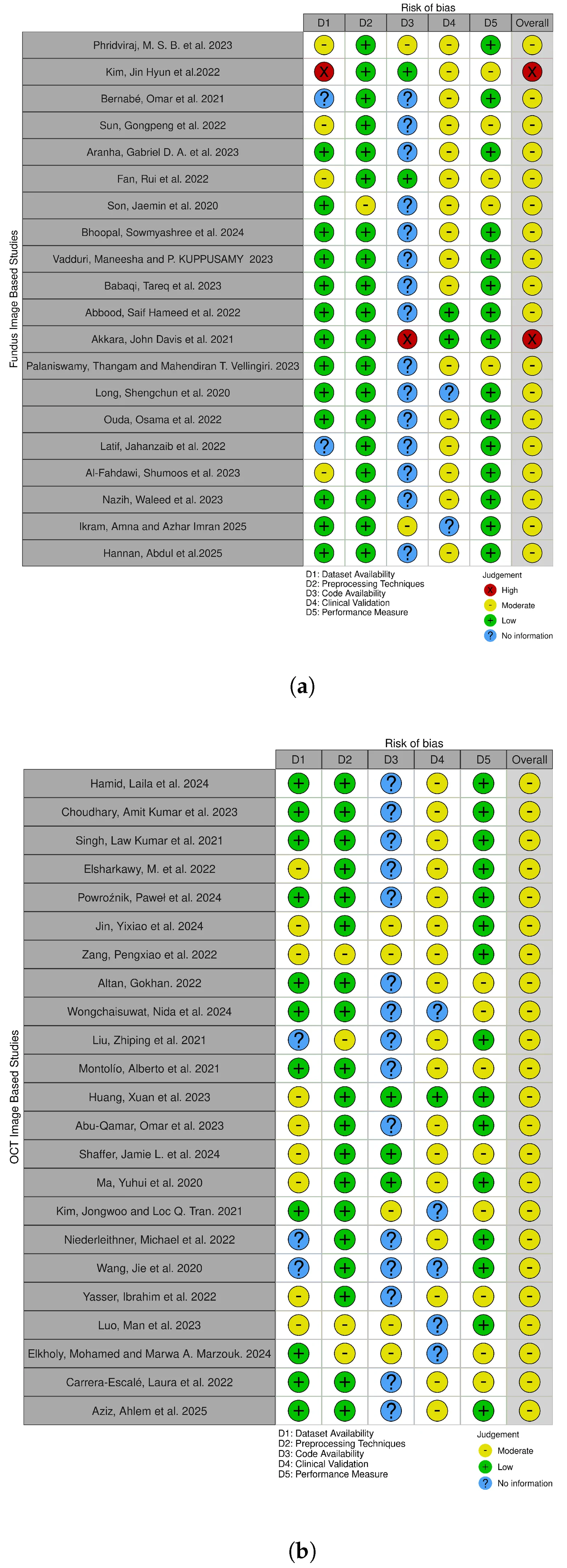
Risk of Bias (RoB) signal plots for included studies: (**a**) fundus-based studies; (**b**) OCT-based studies.

Study-wise ROB assessment for both domains is shown in the Figure 9. These ROB findings for fundus and OCT-based studies directly address RO3 regarding the measurement of ROB in studies under consideration.

### 3.3. Inter-Rater Reliability Agreement

**Figure 10:**
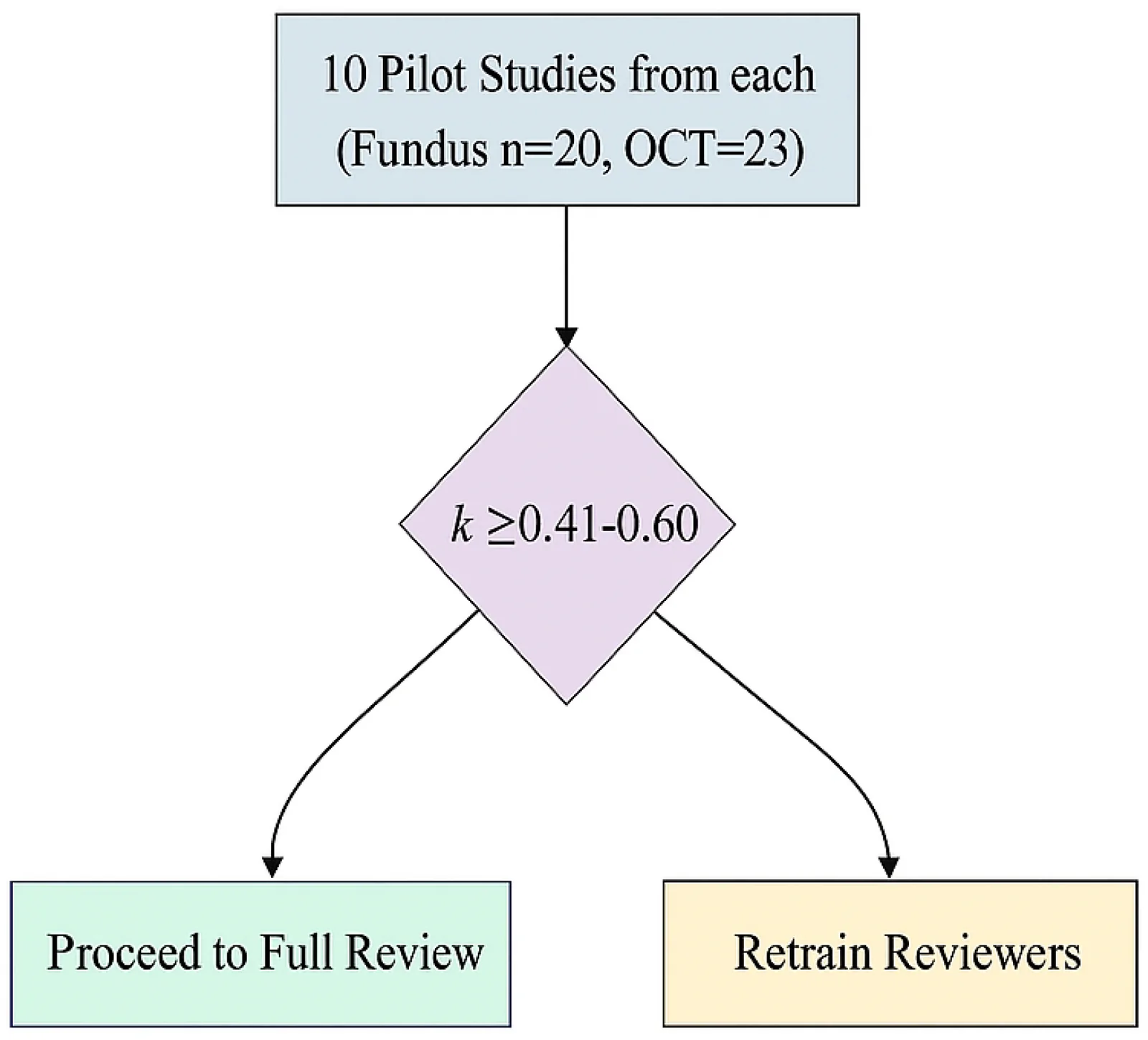
The decision flow for inter-rater reliability (IRR).

The inter-rater reliability (IRR) agreement count between two reviewers is measured by Cohen’s Kappa (k) values based on ten studies as a sample subset. The decision flow for calculating inter-rater reliability is shown in Figure 10.

Unlike manually using formulas, we calculated the Cohen’s Kappa (k) and weighted k via a Python 3.10 (Python Software Foundation, Wilmington, DE, USA). script with the help of the statsmodels library.

The actual ratings of the reviewers are divided into three categories: high risk, low risk, or moderate risk, as shown in Table 10.

The corresponding results of these ratings are shown in Table 11.

The agreement count between two reviewers is 80% (8/10) for both fundus and OCT studies. The IRR has shown moderate to substantial agreement for both modalities. The weighted kappa shows that most of the disagreements are along similar categories for both cases. A full-dataset IRR analysis was not performed, as the initial subset calibration demonstrated consistent reviewer agreement.

### 3.4. Technical Rigor Assessment

**Figure 11:**
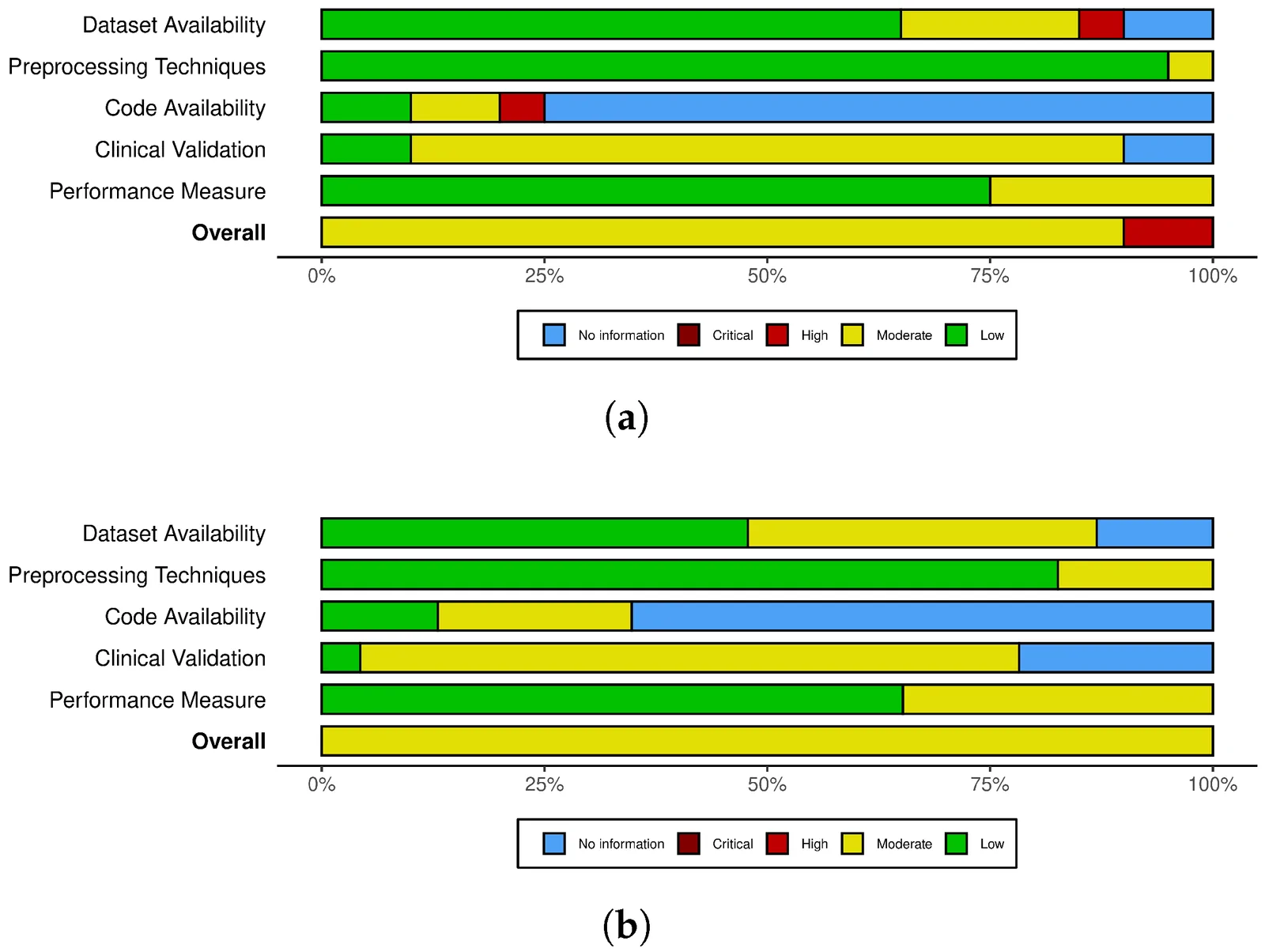
Bar plot for fundus and OCT-based studies: (**a**) fundus-based studies; (**b**) OCT-based studies.

We applied the CLAIM technical compliance tool’s checklist on all selected studies for both modalities and visualized the results via the following bar plots. Figure 11 shows the bar plot of fundus-based studies and the bar plot of OCT-based studies.

From this, we got the key insights presented in the following Table 12.

The compliance rate for included fundus studies varies from 95% (highest) for preprocessing techniques to 10% (lowest) for code availability, clinical validation, and performance analysis, with an overall rating of 75%, which is quite reasonable. This means the preprocessing techniques, dataset availability, and performance analysis are well described in the literature under consideration using different performance parameters; however, most of the existing work didn’t allow code/data sharing for reproducibility of presented results, while most of these lack clinical validations on real-world cases. The studies based on OCT images have shown low compliance rates as compared to fundus-based studies. The domain items dataset’s availability and preprocessing techniques were applied in 47.9% and 82.6% of the studies, respectively. At the same time, code availability and clinical validation lag in OCT-based studies are also showing a low compliance rate. More than half of the studies clearly presented performance analysis of designed frameworks, showing a compliance rate of 65.2%. The OCT-based studies show consistency of a lack of reproducibility and real-world application feasibility.

The data of reviewer agreement were analyzed to estimate the inter-class correlation coefficient (ICC(2,1)) and the corresponding 95% confidence intervals for fundus and OCT-based studies. These analyses were performed using the Python statsmodel library, and the results are summarized in Table 13 below.

The findings indicate that the reviewers demonstrated consistent application of the CLAIM criteria across both domains. However, the inter-rater reliability was lower for OCT-based studies than for fundus-based studies, suggesting stronger agreement among reviewers when evaluating fundus data. Overall, the statistical outcomes and the CLAIM-based technical rigor analysis highlight how variations in reporting practices and the adoption of hybrid methodological approaches can influence the reproducibility of AI studies.

### 3.5. Technical–Clinical Integrity Index (TCII) Outcomes

In order to combine methodological rigor presented in Table 9 and transparency in Table 12 into one comparative measure, every study was graded with a TCII ratings ranging between 0 and 1 as per Equation (3). The mean TCII in all the studies were 0.73 (±0.08) and the values ranged between 0.53–0.94. Fundus based studies were slightly higher (0.75) than OCT based studies (0.71), with better consistent information of the datasets, and better validation reporting.

**Figure 12:**
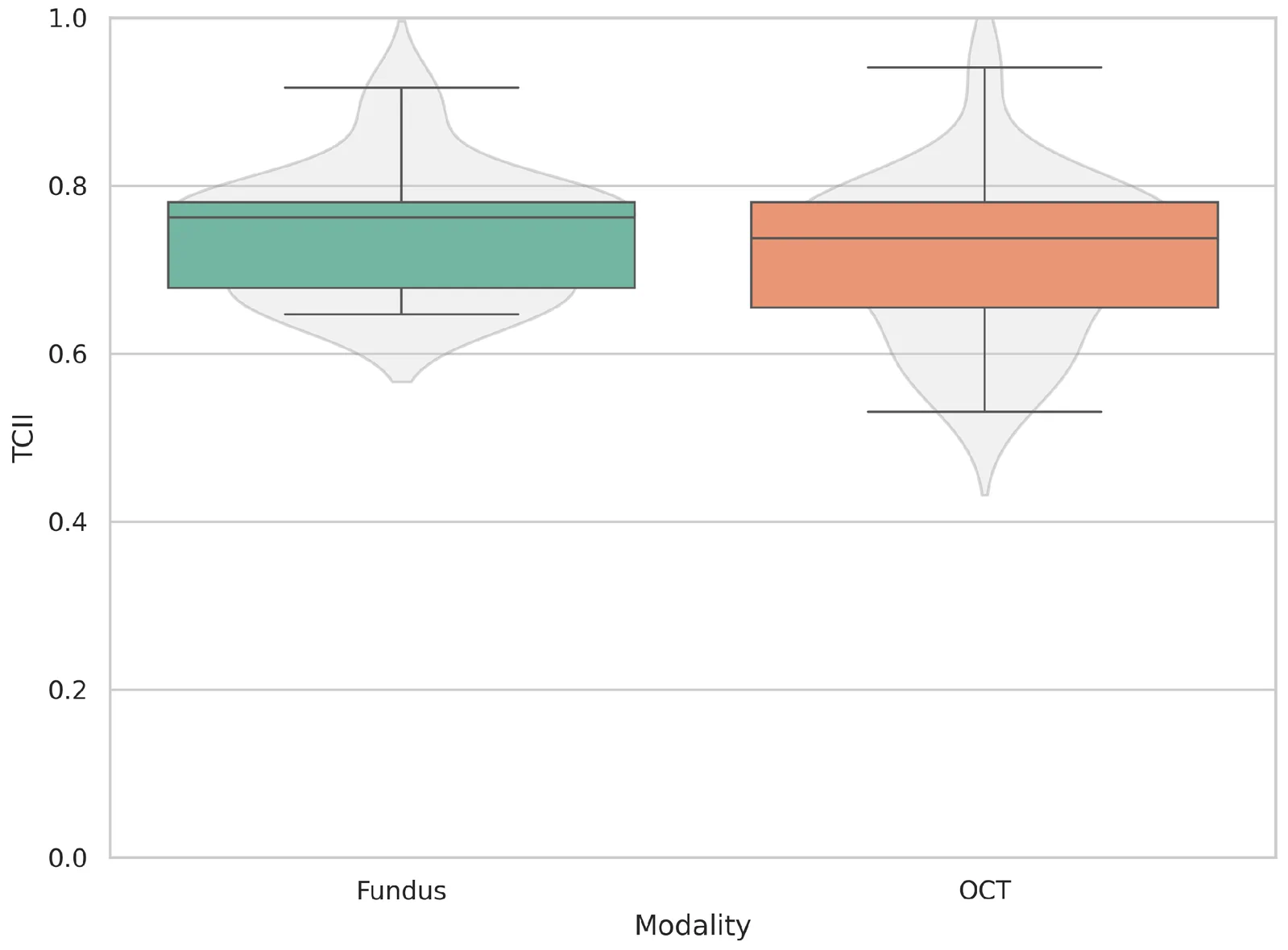
Distribution of Technical–Clinical Integrity Index (TCII) among fundus and OCT.

**Figure 13:**
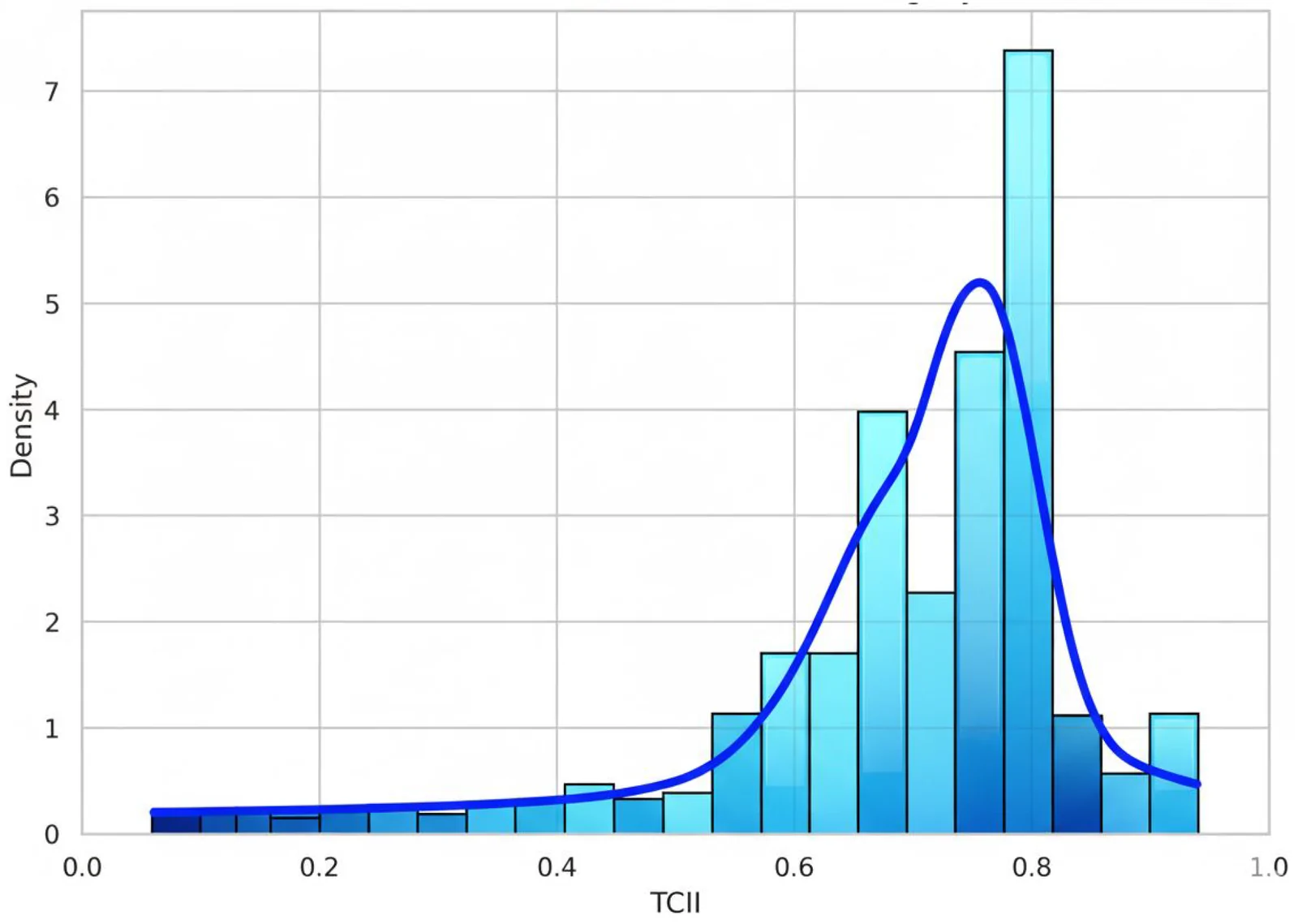
Distribution of Technical–Clinical Integrity Index (TCII) across studies.

The values of TCII distribution by imaging modality can be seen in Figure 12. About 65% of the works belonged to high-integrity (TCII ≥ 0.70) means a larger set had some validation and transparency and publicly or even partially shared data. The rest 35% were considered moderate-integrity (0.50 ≤ TCII < 0.70) with most of them exhibiting a weak code-availability or internal-only validation. None of the studies met the 0.50 or below, meaning that total transparency and reproducibility across the recent literature of retinal AI has achieved a very high and stable ratings. The overall distribution of TCII across studies is shown in the Figure 13.

The correlation analysis did not show a statistically significant relationship between TCII and reported diagnostic accuracy (Spearman ρ = 0.05, *p* = 0.76). This suggests that higher methodological integrity does not necessarily translate into higher reported performance. One likely explanation is the limited variability in accuracy values, as most studies report similarly high performance.

More importantly, this finding highlights that accuracy alone may not reflect clinical readiness. While performance metrics appear saturated, TCII captures additional dimensions such as validation rigor, transparency, and reproducibility, which are critical for reliable clinical translation. Following is the Table 14 showing TCII ratings distribution by studies.

Some of the studies had high methodological integrity with pinpoint high accuracies, whereas most the studies had moderate methodological integrity but equal accuracy, indicating that accuracy is no longer a distinguishing factor in the contemporary research of retinal AI research.

**Figure 14:**
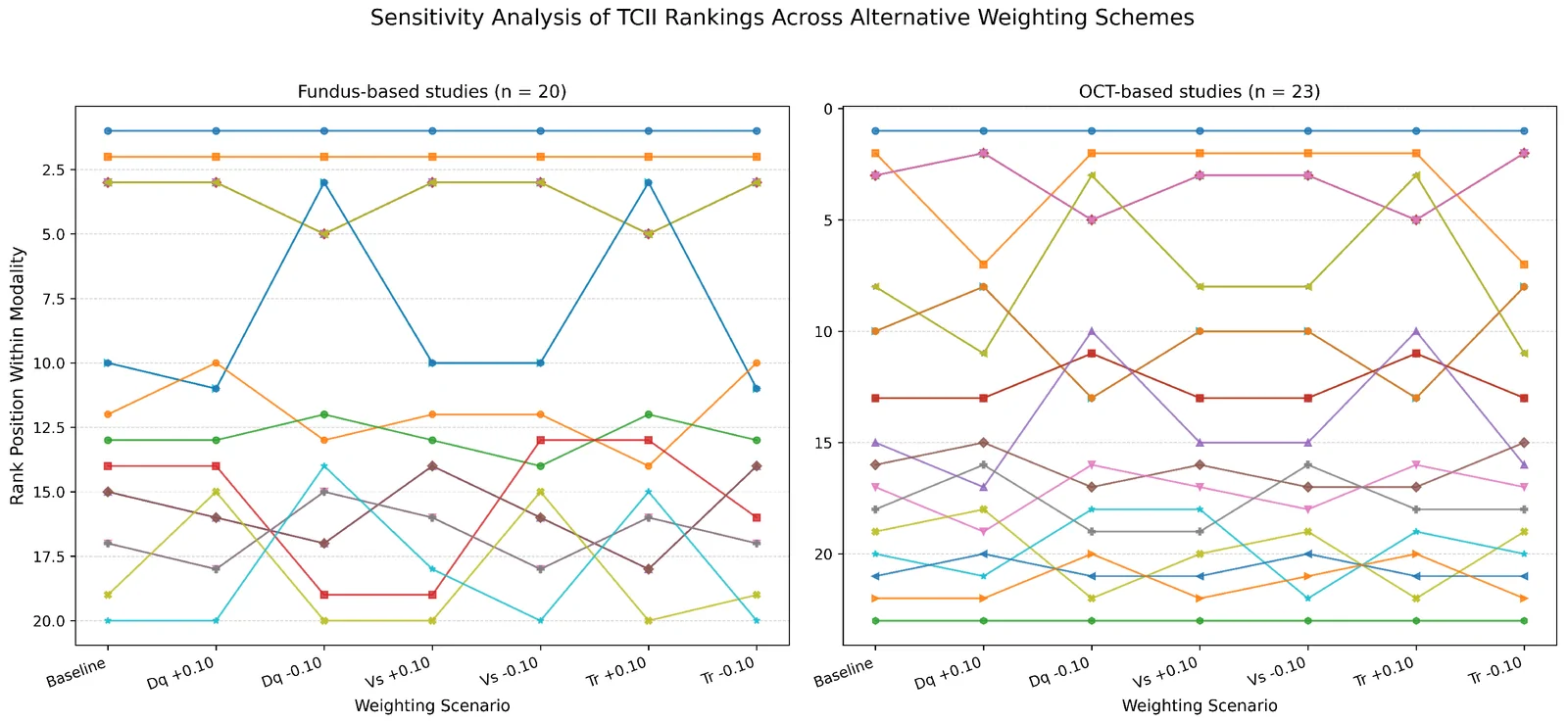
Sensitivity analysis of TCII rankings under alternative weighting schemes for fundus- and OCT-based studies. Each line represents an individual study, and rank positions were recalculated after varying the weights of dataset quality, validation strategy, and transparency/reproducibility. Despite minor fluctuations, the overall ranking structure remains largely stable across all scenarios, supporting the robustness of the TCII framework.

A sensitivity analysis was performed by varying each TCII component weight by ±0.10 while proportionally adjusting the remaining components to preserve a unit-sum constraint. As shown in Figure 14, the rankings of fundus- and OCT-based studies remained largely stable across the tested weighting scenarios, with only minor positional shifts. This indicates that the comparative conclusions derived from TCII are robust to moderate changes in weighting assumptions.

Due to overlapping rank trajectories, multiple studies share identical ranking patterns and are therefore visually represented by the same line.

### 3.6. Analytical Insights Derived from TCII

Several patterns are shown by the TCII analysis. Firstly, methodological integrity was high and the performance measures like accuracy did not vary a lot across studies meaning that most models have reached a performance plateau. Secondly, more clearly than the type of studies or size of datasets, the methodological rigor of the studies remained differentiated by transparency and validation practice. Thirdly, high-TCII studies where the data are publicly released or partially shared, include specific steps of preprocessing, and use some external validation showed more stable and understandable reporting, even when the model performance was comparable to others. Taken together, these results indicate that AI studies in retinal imaging have long since reached the phase of optimization of performance to a phase where reproducibility, interpretation, and the depth of validation become the actual measure of the quality of a study and translation readiness.

### 3.7. Translational Readiness Framework (TRF) Mappings

To place the analytical findings into a wider context of healthcare-analytics, all studies were placed in a Translational Readiness Framework (TRF) made of three hierarchical stages:

- Data Integrity: High dataset quality, diversity, and bias control. (Achieved by approximately 65% of studies with TCII ≥ 0.70).
- Validation Rigor: Evidence of independent or multi-center validation, reproducibility testing, and transparent reporting of preprocessing and evaluation. (Observed in about 25% of studies).
- Clinical Deployment Readiness: Demonstration of interpretability, clinician-in-the-loop evaluation, or limited real-world testing. (Noted in fewer than 10% of studies).

**Figure 15:**
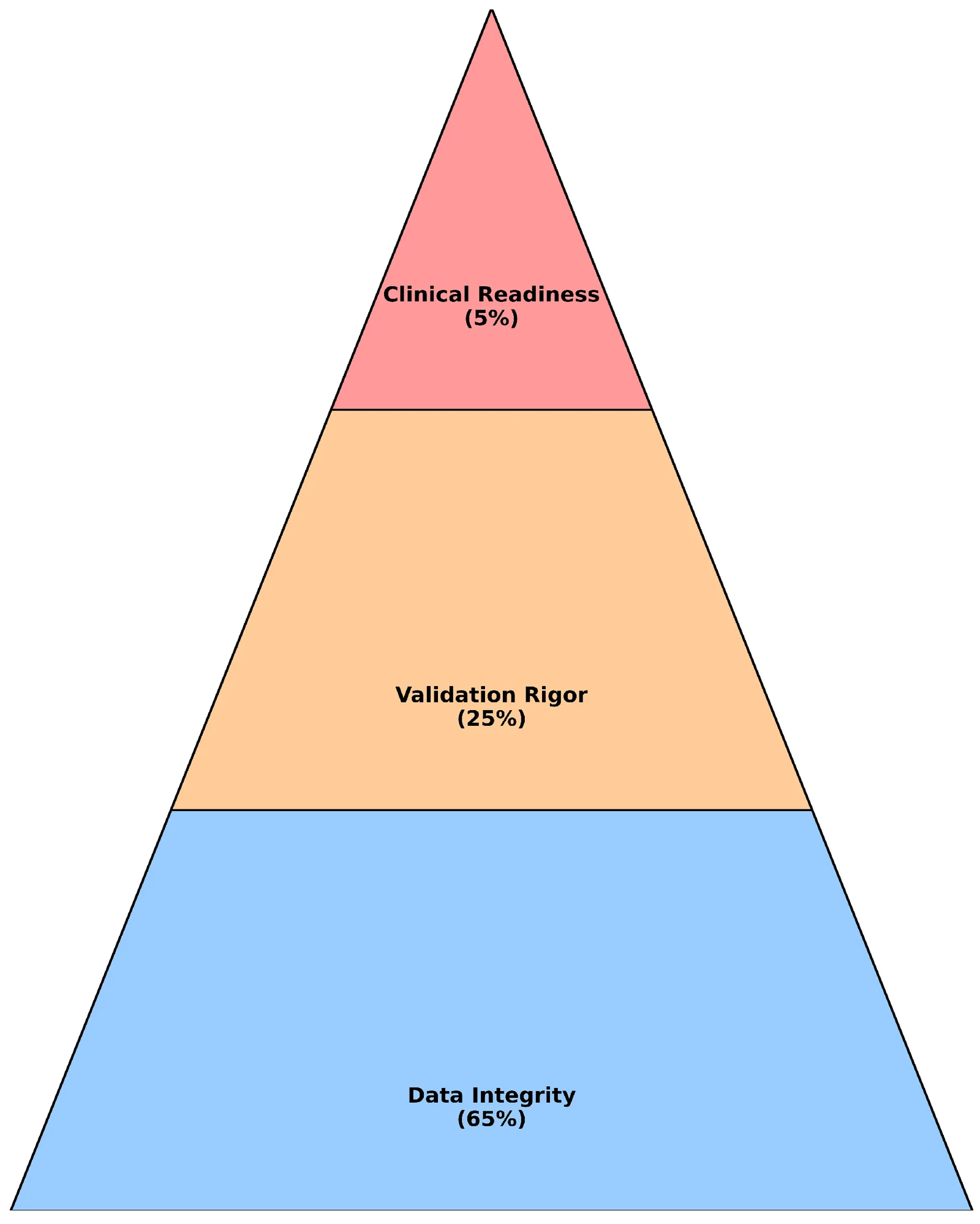
Translational Readiness Framework (TRF) Pyramid.

Figure 15 shows that, applying these indicative thresholds, most studies were consistently mapped to the Data Integrity stage, with only a subset progressing to Validation Rigor and very few meeting criteria suggestive of Clinical Deployment Readiness. This reinforces that advancement across TRF stages is constrained primarily by the lack of external validation and real-world evaluation rather than model performance. Although reproducibility and transparency are mostly attained, but translation into clinical workflows remains limited. Integrating TCII with TRF therefore provides a dual perspective, quantitatively capturing methodological soundness and qualitatively assessing how close each study is to clinical implementation.

## 4. Discussion

### 4.1. Overview of Key Findings

In this review, recent studies in deep learning for the detection and segmentation of retinal diseases using fundus and OCT images that were published between 2020 and 2025 were analyzed. The combination of the *custom AI-specific risk-of-bias (ROB) assessment*, CLAIM, and TCII allows seeing a clear pattern, in which the reported performance is relatively high, but the methodological rigor and clinical validation are quite different. One of the most notable findings is that there is no significant correlation between TCII and the reported accuracy, and the value of the correlation is (rho = 0.05, *p* = 0.76). This implies that the performance metrics are no longer enough to distinguish a well-conducted study from a weakly validated one. In reality, a large number of models have equally high accuracy, yet have significant differences in the way they are designed, in the level of transparency, and reproducibility. This finding is consistent with the previous studies that positively affect the inflated performance and enhance reproducibility of pipelines through well-documented pipelines and open reporting of performance outcomes [16,17,22,100,101,102,103]. Some of the studies that have lower methodological transparency have already reported excellent performance, which suggests that accuracy can be used to mask the weaknesses in the dataset processing or validation approach. This supports the necessity to analyze AI systems on a wider basis, with parameters such as data provenance, labeling clarity, reproducibility, and external validation being considered [104].

These models are still difficult to translate into clinical application, even though there is technical advancement. The application of models in settings other than those in which they were trained is usually associated with deterioration of their performance due to variations in imaging devices, acquisition protocols, and patient groups of the models used in the studies and research studies are prone to failure in their new settings of application [105]. The generalization is further complicated by device heterogeneity between fundus cameras and OCT systems, which often necessitates local adaptation to be made [106]. Moreover, inconsistency in expert annotations creates uncertainty in ground truth labels, and this can be transferred during training and evaluation. There are also practical barriers, such as the ability to integrate into clinical workflows, clinician trust, and point of care interpretability, that remain in place and persistently hinder progress in this area of implementation and adoption [107,108]. The regulatory requirements create an additional complexity since AI systems are to be proven to be safe, stable and clinically beneficial to technical performance [109].

Diversity between types of diseases also affects the maturity of the methodology. The results of the studies regarding diabetic retinopathy are more likely to demonstrate uniform performance due to the presence of extensive datasets of patients and the grading criteria. Conversely, glaucoma studies are usually predisposed to issues pertaining to the determination of reproducible ground truth, which increases variability. Heterogeneous imaging protocols and presentation of the disease affect the studies of AMD, and the RVO is not explored sufficiently, which restricts comparability. These disparities indicate that the quality of the datasets, practice of annotation, and clinical consensus are highly related to translational readiness, and not just model architecture itself [105,106,107,108].

### 4.2. Translational Readiness Framework (TRF): Implications for Practice

In order to provide a more relevant connection between the methodological quality and clinical applicability, the Translational Readiness Framework (TRF) is introduced in this review. Placing the included studies in this framework, the majority of them are at the data integrity stage, fewer of them are at the robust validation stage, and a small number of them are at the clinical readiness stage. This is an imbalance of the larger disparity between technical development and real-world adoption [110,111,112]. All the stages of the TRF are associated with practical requirements. To go beyond data integrity, there should be clear documentation of the datasets, and it should not take shortcuts in methods like data leakage. The next stage of the rigor of validation requires extraneous testing and reproducibility in separate cohorts. Clinical readiness requires a higher standard of evidence, such as multicenter assessment, comparison with clinical standards and determination in actual workflows. Such expectations are in line with the present guidelines, like CONSORT-AI and SPIRIT-AI [48].

Despite its structured design, the TRF is to be viewed as a systematic guideline, but not a strict set of regulations. The important aspects like regulatory approval routes, long-term monitoring and lifecycle management were not always found in the literature and thus could not be systematically included. In this sense, the TRF is mainly used in pointing out the gaps that need to be covered prior to clinical implementation. From a healthcare analytics perspective, the TRF can be used as a decision-support checklist: analytics teams may use TCII-derived evidence to prioritize models for pilot testing or procurement, while clinical researchers can identify which methodological aspects require reinforcement before translation into practice. The synergy of TCII and TRF results is that standardized protocols of validation and open data management are required. Open reporting of dataset provenance, validation workflows and code repositories should be mandatory for journals and funding bodies. The use of AI tools in hospitals must involve the implementation of TCII kind of evaluation in their assessment pipelines, and the procurement decisions should take into consideration the reproducibility and not the accuracy.

Previous reviews on retinal AI have presented detailed analysis of architectures and datasets but have been largely descriptive and concentrated on performance summaries as opposed to synthesizing their methodology. In comparison, the TCII-based analysis in the given research measures the quality of methodology. This methodology fills the gap between descriptive and analytical, providing a more concrete idea about the influence of reproducibility and depth of validation on the practicality of real-world problems [19,105,113,114,115]. Overall, the combination of TCII and TRF is an effective approach to go beyond the mere summaries and concentrate on what can be helpful in the clinic using AI systems. The next step of the work would be to make the reporting more standardized, enhance the transparency of the datasets, and involve technical developers and clinical stakeholders more closely.

### 4.3. Limitations

These findings have a number of limitations that should be taken into consideration in interpreting them. Only English-language studies have been included and this could have omitted the opportunity to include the relevant work published in other languages; hence, this could have influenced the global representation of the evidence. Besides this, the fact that the review was limited to single-modality studies also limits the generalizability of the findings to new multi-modal methods. The publication bias was not formally evaluated because of high heterogeneity in datasets, measures of the evaluation and reporting. This leads to the fact that reporting bias cannot be ruled out. The ROB framework applied in the present review is deliberately simplified, and it is based on five domains, which represent the major areas of transparency, validation, and reproducibility. Although this enhances interpretability, it fails to capture all the sources of bias that may arise, including labeling variability or patient selection. Inter-rater reliability was evaluated on a representative sample of the studies to ensure that the same is consistent before full evaluation. The TCII is founded on an expert judgment weighting scheme as opposed to empirical optimization. Even though sensitivity analysis revealed that rankings did not change significantly when moderate variations were applied, additional reinforcement by data-driven methods would be beneficial to the framework. Moreover, explainability and regulatory requirements were not directly considered since the reporting was not consistent in all studies. Lastly, TCII was not correlated with accuracy, indicating that performance measures commonly reported might not be a good indicator of translation readiness, which means that more comprehensive evaluation criteria are required.

## 5. Conclusions

This review is based on the recent deep learning methods of retinal disease detection based on fundus and OCT imaging. The combination of PRISMA 2020, the custom AI-specific risk-of-bias (ROB) assessment, CLAIM, and a new Technical-Clinical Integrity Index (TCII) provided the study with a more in-depth perspective of the role of methodological quality on the reliability of reported findings. In the included studies, high diagnostic performance was observed, although not always indicative of good validation practices and reproducibility. The models built with the help of various and well-documented datasets were more likely to be robust, and the weaknesses in transparency and external validation were still prevalent. These methodological differences can be quantitatively captured by the TCII and put in a larger context of clinical processes by the TRF. Collectively, all these tools emphasize the fact that the advancement of retinal AI is not restrained by the ability of an algorithm anymore but by the quality of validation, reporting, and real-world testing. Future research should apply these analytical tools prospectively across multi-center studies and additional imaging modalities to accelerate safe, evidence-based integration of AI into ophthalmic care.

## Figures and Tables

**Table 1:** Description of research objectives.

Research Objectives	Research Questions	Motivation	Outcome
RO1	Which type of DL models are used for retinal disease detection/segmentation for a single image modality in the last six years (2020–2025)?	To investigate the diagnostic approaches commonly used in practice for eye image analysis and disease detection.	A summary table of the most commonly used ML/CNN/hybrid techniques for fundus and OCT-based domains, serving as baseline input for later TCII diagnostic reliability assessment.
RO2	What types of datasets are commonly used, and how do they impact the diagnostic performance of applied models?	To investigate the impact of datasets on diagnostic validity due to varying quality and sizes.	A summary of public/private/hybrid datasets with the total number of image samples for fundus and OCT-based domains, including dataset-specific parameters incorporated into TCII diagnostic validity ratings.
RO3	How can the risk of bias be measured in current studies using a ROB measurement tool to ensure diagnostic trustworthiness?	To evaluate the methodological soundness and clinical reliability of the presented studies.	Quantitative categorization of studies as low, moderate, or high risk-of-bias ratings, numerical inputs contributing to TCII diagnostic trustworthiness scoring.
RO4	Have the selected studies used proper reporting practices of the different parameters commonly used to support reproducible medical diagnosis?	To assess how well the selected literature incorporates key AI-related parameters influencing diagnostic interpretability and reproducibility.	Application of the CLAIM criteria on selected items, reporting-quality ratings included in TCII computation.
RO5	How can methodological quality, validation rigor, and transparency be integrated into a unified analytical measure reflecting overall diagnostic reliability?	To combine findings from RO1–RO4 into a single interpretive metric representing clinical diagnostic robustness.	Development of the TCII that merges dataset quality, validation strategy, and reporting transparency for each study, reflecting diagnostic readiness.
RO6	How can synthesized findings guide clinical diagnostic decision-making for AI adoption in ophthalmology?	To translate analytical insights into a practical framework supporting safe clinical deployment of diagnostic AI systems.	Formulation of a TRF linking technical rigor (TCII ratings) with stages of data integrity, validation, and clinical diagnostic implementation.

**Table 2:** Description of inclusion and exclusion criteria.

Criterion	Sr. No.	Description
Inclusion	1	Only single modality eye image datasets (private/public, fundus/OCT) and use of non-invasive methods, ML/DL techniques, mainly on DR/RVO/AMD and glaucoma were included.
	2	Studies conducted between January 2020 and August 2025.
	3	Only studies published in English.
	4	Studies reporting standard performance metrics.
	5	Report at least one diagnostic performance metric (sensitivity, specificity, AUC, accuracy, precision, recall, and F1-score).
Exclusion	1	Research on eye disease diagnosis using invasive techniques.
	2	Studies conducted before January 2020.
	3	Multi-modal frameworks and datasets.
	4	Non-English studies.
	5	Studies involving animal eye image datasets.

**Table 3:** Description of the PICO strategy.

Element	Description
Population (P)	Patients with retinal diseases (DR, RVO, and AMD), represented by fundus and OCT images.
Intervention (I)	DL models for prediction and classification of retinal diseases.
Comparator (C)	Other DL models, traditional image analysis approaches, or expert grading.
Outcomes (O)	Performance metrics (accuracy, sensitivity, specificity, AUC, and F1-score) and methodological details (dataset type, model architecture, and augmentation strategy).

**Table 4:** Attribution of screening stages between automation tools and human reviewers.

Screening Stage	Responsible Agent(s)
Initial title/abstract prioritization	Rayyan, Elicit.ai, and Bohrium (parallel scoring)
Threshold calibration	Pilot testing on a random subset of 100
Records scoring ≥ 70	Advanced to human screening
Records scoring < 70	Provisionally excluded
Quality control check	20% random sample manually re-screened
Title/abstract screening	Two independent human reviewers
Conflict resolution	Third reviewer adjudication
Full-text screening	Two independent human reviewers
Final inclusion	Consensus decision

**Table 5:** Domain items for custom AI-specific ROB calculation.

Domain Item	Description	Rationale for Inclusion
D1	Dataset availability	Transparency of datasets enables independent verification, external validation, and assessment of population representativeness, which are essential for reducing selection and reporting bias.
D2	Preprocessing techniques	Inadequately reported preprocessing steps may introduce hidden data leakage or model optimization bias, directly affecting reproducibility and performance reliability.
D3	Code availability	Access to source code supports reproducibility, facilitates technical scrutiny, and reduces implementation bias arising from undocumented model configurations.
D4	Clinical validation (overall validity of the external real-world datasets)	Absence of external clinical datasets increases the risk of overfitting and limits the translational applicability of reported results.
D5	Performance measure	Selective or non-standard reporting of performance metrics may inflate perceived effectiveness and hinder fair comparison across studies.

**Table 6:** Custom AI-specific risk-of-bias (ROB) assessment with signaling questions.

Rating	D1	D2	D3	D4	D5
Low risk	If publicly available, well-documented, and allows replication?	If all steps are clearly described and appropriate?	If code is fully available (GitHub/link) and reproducible?	If the model is validated on real-world external data?	If appropriate and clearly defined?
High risk	If unavailable, proprietary, or selectively shared?	If steps are poorly described or inappropriate?	If code unavailable, replication impossible?	If no validation beyond the training dataset?	If metrics are inappropriate or misleading?
Moderate risk	If partially available or requires special access?	If some steps are described, but details are missing?	If partial code is available, but insufficient for replication?	If limited validation (only internal CV or small external samples)?	If reported but lacking justification or incomplete?
No information	If no details on dataset availability?	If no description of preprocessing steps?	If no mention of code availability?	If no details on clinical validation?	If no metrics reported?

**Table 7:** Comparison of studies based on fundus image datasets, methodology, tasks and performance.

Feature/Study	[51]	[52]	[53]	[54]	[55]	[56]	[57]	[58]	[59]	[60]
Year	2023	2022	2021	2022	2023	2022	2020	2024	2023	2023
Dataset (Public)	✓		✓					✓	✓	✓
Dataset (Private)		✓		✓						
Dataset (Hybrid)					✓	✓	✓			
Sample Size (s)	36,739	102,237	1047	4574	40,027	82,852	104,875	3662	3049	4200
Model: Only CNN (s)			✓	✓	✓					✓
Model: Hybrid	✓	✓				✓	✓	✓	✓	
D1	—	✗	?	—	—	—	✓	✓	✓	✓
D2	✓	✓	✓	✓	✓	✓	—	✓	✓	✓
D3	—	✓	?	?	?	✓	?	?	?	?
D4	—	—	—	—	—	—	—	—	—	—
D5	✓	—	✓	—	✓	—	—	✓	✓	✓
Disease focus	DR ([51,58]); Multi-disease ([52,53,54,55,57,59,60]); Glaucoma ([56])									
Primary task	Grading ([51]); Classification ([52,53,55,56,58,60]); Detection ([54,57]); Seg. + Classification ([59])									
Accuracy (%)	96.8	94.3	99.9	97.1	99.1	95.0	99.9	98.0	98.3	84.0
Feature/Study	[61]	[62]	[63]	[64]	[65]	[66]	[67]	[68]	[8]	[69]
Year	2022	2021	2023	2020	2022	2022	2023	2023	2025	2025
Dataset (Public)	✓	✓	✓	✓	✓	✓	✓		✓	✓
Dataset (Private)										
Dataset (Hybrid)								✓		
Sample Size (s)	1200	1113	1744	163	3200	958	10,000	1923	3669	38,777
Model: Only CNN (s)	✓				✓	✓				✓
Model: Hybrid		✓	✓	✓			✓	✓	✓	
D1	✓	✓	✓	✓	✓	?	—	✓	✓	✓
D2	✓	✓	✓	✓	✓	✓	✓	✓	✓	✓
D3	?	✗	?	?	?	?	?	?	—	?
D4	✓	✓	—	?	—	—	—	—	?	—
D5	✓	✓	—	✓	✓	✓	✓	✓	✓	✓
Disease focus	DR ([8,61,63,64,68,69]); Glaucoma ([62,66]); Multi-disease ([65,67])									
Primary task	Classification ([61,62,65,66,67,68,69]); Detection ([64]); Seg. + Classification ([63]); Grading ([8])									
Accuracy (%)	93.6	95.6	99.1	87.0	94.3	95.7	99.8	82.5	93.0	83.2

Notes: (1) (✓) indicates presence of a feature; (2) (✗) absence of a feature; (3) (—) partial information; (4) (?) no information.

**Table 8:** Comparison of studies based on OCT image datasets, methodology, tasks and performance.

Feature/Study	[70]	[71]	[72]	[73]	[28]	[29]	[74]	[75]	[30]	[31]	[76]	[32]
Year	2024	2023	2021	2022	2024	2024	2022	2022	2024	2021	2021	2023
Dataset (Public)	✓	✓			✓	✓		✓				
Dataset (Private)				✓			✓		✓	✓	✓	✓
Dataset (Hybrid)			✓									
Sample Size (s)	84,495	84,484	140	188 Volumes	84,495	1103	626	62,617	120	246	294	108,312
Model: Only CNN (s)		✓					✓	✓				
Model: Hybrid	✓		✓	✓	✓	✓			✓	✓	✓	✓
D1	✓	✓	✓	—	✓	—	—	✓	✓	?	✓	—
D2	✓	✓	✓	✓	✓	✓	—	✓	✓	—	✓	✓
D3	?	?	?	?	?	—	—	?	?	?	?	✓
D4	—	—	—	—	—	—	—	—	?	—	—	✓
D5	✓	✓	✓	✓	✓	✓	✓	—	—	✓	—	✓
Disease focus	AMD ([70]); Glaucoma ([72]); DR ([29,31,73]); ME ([75]); Multi-disease ([28,30,32,71,74,76])											
Primary task	Classification ([28,31,32,70,71,75,76]); Detection ([72,73]); IQA ([29]); Seg. + Detection ([30,74])											
Accuracy (%)	98.8	99.2	97.0	96.9	98.9	90.3	80.0	99.2	95.5	82.0	87.7	99.0
**Feature/Study**	[77]	[33]	[78]	[79]	[80]	[81]	[82]	[83]	[84]	[85]	[86]	
Year	2023	2024	2020	2021	2022	2020	2022	2023	2024	2022	2025	
Dataset (Public)			✓	✓					✓	✓	✓	
Dataset (Private)	✓	✓			✓	✓	✓					
Dataset (Hybrid)								✓				
Sample Size (s)	144	940	229	109,309	9 volumes	1676	91 Patients	192,000 Pairs	35,468	726	24,000	
Model: Only CNN (s)	✓	✓	✓	✓				✓	✓	✓		
Model: Hybrid					✓	✓	✓				✓	
D1	—	—	—	✓	?	?		—	—	—✓	✓	
D2	✓	✓	✓	✓	✓	✓	✓	—	—	✓	✓	
D3	?	✓	✓	—	?	?	?	—	✓	?	?	
D4	—	—	—	?	—	?	—	?	✓	—	—	
D5	✓	—	✓	—	✓	✓	—	✓	—	—	✓	
Disease focus	DR ([80,82,85,86]); Glaucoma ([83]); Multi-disease ([33,77,78,79,81,84])											
Primary task	Classification ([79,82,84,85]); Segmentation ([33,78,81,86]); Image Enhancement ([77,80,83]);											
Accuracy (%)	?	?	93.9	98.7	?	?	94.4	?	97.0	?	98.0	

Notes: (1) (✓) indicates presence of a feature; (2) (—) partial information; (3) (?) no information.

**Table 9:** Domain-wise ROB analysis.

Study	Domain Item	Low Risk (%)	High Risk (%)	Moderate Risk (%)	No Information (%)
	D1	65	5	20	10
	D2	95	0	5	0
Fundus	D3	10	5	10	75
	D4	10	0	80	10
	D5	75	0	25	0
	D1	47.9	0	39.1	13
	D2	82.6	0	17.4	0
OCT	D3	13.1	0	21.7	65.2
	D4	4.3	0	73.9	21.7
	D5	65.2	0	34.8	0

**Table 10:** ROB ratings across studies by two reviewers.

Study Type	Study	Reviewer 1	Reviewer 2
	[51]	Moderate	Moderate
	[52]	High	High
	[53]	Moderate	Moderate
	[54]	Moderate	Moderate
Fundus	[59]	Moderate	Moderate
	[60]	High	Moderate
	[62]	High	High
	[64]	Moderate	Moderate
	[65]	High	Moderate
	[68]	Moderate	Moderate
	[70]	Moderate	Moderate
	[71]	Moderate	Moderate
	[73]	High	Moderate
	[76]	Moderate	Moderate
OCT	[33]	Moderate	Moderate
	[32]	Moderate	Moderate
	[78]	Moderate	Moderate
	[80]	Moderate	High
	[81]	High	High
	[85]	Moderate	Moderate

**Table 11:** Agreement statistics between reviewers.

Study Type	Reviewer 1/Reviewer 2	Low	High	Moderate	Cohen’s k	Agreement
Fundus	Low	0	0	0	0.524 (95%, CI: 0.28–0.97)	8/10
	High	0	2	1		
	Moderate	0	1	6		
OCT	Low	0	0	0	0.515 (95%, CI: 0.30–0.98)	8/10
	High	0	1	1		
	Moderate	0	1	7		

**Table 12:** Compliance checklist.

CLAIM Items (Grouped)	Compliance (%) Fundus-Based Studies	Compliance (%) OCT-Based Studies
Dataset Availability	65	47.9
Preprocessing Techniques	95	82.6
Code Availability	10	13.1
Clinical Validation	10	4.3
Performance Analysis	75	65.2

**Table 13:** Summary of Inter-Rater Reliability (ICC) between Reviewers for fundus and OCT-Based Studies.

Study Type	ICC (2,1)	95% CI	Mean Diff.	Max. Diff.
Fundus	0.911	[0.896, 0.923]	2.3	3
OCT	0.643	[0.422, 0.792]	3.0	5

**Table 14:** Transparency and Computational Integrity Index (TCII) Ranges, Number of Studies, Mean Accuracy and Typical Characteristics.

TCII Range	No. of Studies (n = 43)	Mean Accuracy (%) ± SD	Typical Characteristics
0.70–1.00	28 (65%)	96.2 ± 2.4	A larger set had some validation and transparency, publicly available or partially shared datasets, clear preprocessing, consistent reporting.
0.50–0.69	15 (35%)	95.4 ± 3.1	Internal validation, limited code availability, moderate transparency.
<0.50	0 (0%)	–	None in this range; overall reproducibility and transparency were strong.

## Data Availability

The study-level TCII scores, risk-of-bias (ROB) assessments, and sensitivity analysis datasets are publicly available on the Open Science Framework (OSF) at: https://osf.io/m7ba6/ (accessed on 26 April 2026).

## Appendix A. Full Electronic Search Strategies

This appendix reports the complete search strategies used for each database, in accordance with PRISMA 2020 Item 7. All searches were executed independently for each platform using database-specific syntax. The final search across all sources was conducted on 30 August 2025.

### Appendix A.1. PubMed (MEDLINE)

**Last search date:** 30 August 2025

(

  ("Retinal Diseases/diagnosis"[MeSH] OR "Eye Diseases"[MeSH]

   OR "Diabetic Retinopathy"[MeSH]

   OR "Macular Degeneration"[MeSH]

   OR glaucoma[Title/Abstract]

   OR retinal disease*[Title/Abstract])

)

AND

(

  "Artificial Intelligence"[MeSH]

  OR "Machine Learning"[MeSH]

  OR "Deep Learning"[Title/Abstract]

  OR "Convolutional Neural Network*"[Title/Abstract]

)

AND

(

  fundus[Title/Abstract]

  OR "optical coherence tomography"[Title/Abstract]

  OR OCT[Title/Abstract]

)

Filters applied:

- Publication dates: 1 January 2020 to 30 August 2025
- Language: English

### Appendix A.2. IEEE Xplore

**Last search date:** 30 August 2025

(

  ("retinal disease" OR "diabetic retinopathy"

   OR glaucoma OR "macular degeneration")

  AND

  ("machine learning" OR "deep learning"

   OR "convolutional neural network*"

   OR "artificial intelligence")

  AND

  (fundus OR "optical coherence tomography" OR OCT)

)

Refined by:

- Content type: Journals and Conferences
- Publication years: 2020-2025
- Language: English

### Appendix A.3. Scopus

**Last search date:** 30 August 2025

TITLE-ABS-KEY (

  ("retinal disease*" OR "diabetic retinopathy"

   OR glaucoma OR "age-related macular degeneration")

  AND

  ("machine learning" OR "deep learning"

   OR "artificial intelligence" OR CNN*)

  AND

  (fundus OR "optical coherence tomography" OR OCT)

)

AND (PUBYEAR > 2019)

AND (LANGUAGE = "English")

### Appendix A.4. Web of Science Core Collection

**Last search date:** 30 August 2025

TS = (

  ("retinal disease*" OR "diabetic retinopathy"

   OR glaucoma OR "macular degeneration")

  AND

  ("machine learning" OR "deep learning"

   OR "artificial intelligence")

  AND

  (fundus OR "optical coherence tomography" OR OCT)

)

Refined by:

- Document types: Article, Proceedings Paper
- Timespan: 2020-2025
- Language: English

### Appendix A.5. Semantic Scholar

**Last search date:** 30 August 2025

("retinal disease" OR "diabetic retinopathy"

 OR glaucoma OR "macular degeneration")

AND

("machine learning" OR "deep learning"

 OR "artificial intelligence")

AND

(fundus OR OCT)

Year range: 2020-2025

### Appendix A.6. Google Scholar

**Last search date:** 30 August 2025

allintitle:

("retinal disease" OR "diabetic retinopathy" OR glaucoma)

("machine learning" OR "deep learning")

(fundus OR OCT)

Custom range: 2020-2025

### Appendix A.7. Manual Search

The reference lists of all included studies and relevant review articles were manually screened to identify additional eligible studies not captured by electronic database searches.
